# Limitations of boronate affinity chromatography for the specific enrichment of fructose-derived early glycation products in protein analytics

**DOI:** 10.1007/s00216-025-06044-2

**Published:** 2025-08-07

**Authors:** Sebastian Lux, Clara Vogt, Milena Voll, Ralf Hoffmann

**Affiliations:** 1 Institute of Bioanalytical Chemistry, Faculty of Chemistry, Universität Leipzig, Leipzig, Germany; 2 Center for Biotechnology and Biomedicine, Universität Leipzig, Deutscher Platz 5, 04103 Leipzig, Germany

**Keywords:** Boronate affinity chromatography (BAC), Fructation, Amadori and Heyns peptides, Hexose/hexitol-lysine, Multiple reaction monitoring (MRM)

## Abstract

Protein glycation, or non-enzymatic glycosylation, refers to the reaction of reducing sugars with amino groups in proteins to form Amadori and Heyns products for aldoses (e.g., glucose) and ketoses (e.g., fructose), respectively. While Amadori peptides have been well studied after enrichment by boronate affinity chromatography (BAC), it is often assumed that BAC also enriches the isomeric Heyns peptides, although the binding of Heyns rearrangement products seems unlikely due to the very low content of 1,2- and 1,3-*cis*-diols in their dominant tautomeric forms. For seven different tryptic peptide sequences derived from human plasma digests, we showed that the synthetic glucose-modified Amadori peptides can be enriched by BAC with high recovery rates, while the corresponding fructose-modified Heyns peptides did not bind, independent of the buffers and pH used. Reduction of the carbonyl groups with borohydride, yielding the corresponding hexitol-modified peptides, allowed enrichment of both the former Amadori and, more importantly, Heyns peptides.

**Graphical Abstract:**

Molecular interactions of boronic acids with unreduced and reduced Amadori (ARPs) and Heyns rearrangement products (HRPs)

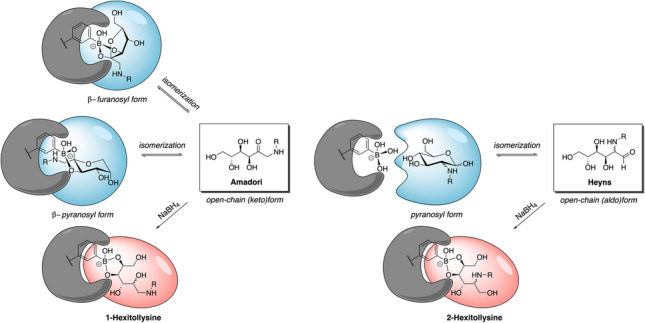

**Supplementary Information:**

The online version contains supplementary material available at 10.1007/s00216-025-06044-2.

## Introduction

Daily consumption of fructose has reached alarming levels due to the marketing of high-fructose corn syrup (HFCS) as a low-cost sweetener. Its excessive consumption has been linked to several lifestyle diseases, including type 2 diabetes, non-alcoholic fatty liver disease (NAFLD), certain cancers, and cardiovascular and kidney diseases [1, 2]. Some detrimental effects of fructose and its metabolites may relate to their ability to form adducts with proteins, lipids, and DNA in a non-enzymatic reaction called glycation, also known as non-enzymatic glycosylation. In this reaction, amino groups condensate with reducing sugars, i.e., aldoses and ketoses, to form labile α-hydroxyaldemines and -ketimines (Schiff bases), which can rearrange to more stable 1-amino-1-deoxy-2-ketoses (Amadori rearrangement products, ARP) and 2-amino-2-deoxy-1-aldoses (Heyns rearrangement products, HRP), respectively. Due to its high prevalence and its implication in diseases characterized by chronically elevated blood glucose levels, glucose glycation (*glucation*) has been addressed in numerous studies, highlighting the relevance of glucose-derived protein adducts in clinical applications. In the context of increased exogenous uptake and the potential for non-negligible endogenous fructose formation [3], post-translational modification of proteins by fructose (*fructation*) in vivo, more specifically HRPs, is receiving increasing attention [4]. Although fructose is present at low micromolar concentrations in the peripheral circulation [5, 6], it is suspected to have a higher glycation potential than glucose due to a higher content of the open (acyclic) tautomeric form (0.5% at 20 °C vs. 0.004% at 30 °C for glucose) [7, 8]. Fructation appears to lead to higher levels of reactive carbonyls and presumably harmful advanced glycation end products (AGEs) [9–11]. As a possible precursor and first stable adduct of the fructation reaction, the specific detection of protein-bound HRPs would meet the high demand for a circulating biomarker representing the effects of temporarily high fructose concentrations [12], which escape the otherwise efficient intestinal and subsequent hepatic fructose metabolism [13]. Due to their extended half-life, site-specific modification levels on proteins could reflect transient increased fructose fluctuations over extended periods in plasma, urine, or other matrices, e.g., as a result of a fructose-rich diet or enhanced polyol pathway activity [5, 14–16].

Despite considerable progress in the analysis of glycation products, studies of Heyns products are still limited because analytical techniques to distinguish between isomeric ARPs and HRPs are scarce. Assessing their individual contributions to glycation has been described as “one of the most intriguing challenges of analytical chemistry applied to the Maillard reaction” [17]. Therefore, both glycation products are typically quantified together as “Amadori/Heyns products” [18]. Highly specific antibodies [19] and mass spectrometry targeting characteristic fragmentation patterns can provide valuable insight into isomeric composition after acid hydrolysis at the amino acid level [16, 20] or digestion at the peptide level [21], allowing analysis of individual protein sites. Although efficient for in vitro experiments, difficulties arise in complex matrices such as plasma with typically low glycation levels. Therefore, enrichment of glycation products is a prerequisite for analyzing glycated proteins by LC–MS, e.g., by boronate affinity chromatography (BAC) [22–24], which is based on the reversible covalent interaction of boronic acids with *cis*−1,2- or 1,3-diols to form five- or six-membered cyclic boronate esters, respectively [25]. Retention is highly pH and buffer dependent [26], allowing binding under alkaline conditions and elution with either acids or strong binding competitors (d-sorbitol) [27]. The potential of BAC to specifically enrich glycated hemoglobin (gHb) was recognized early and has been used in clinical assays for the assessment of gHb as a diabetes biomarker since 1995 [28–30]. Despite the rise of immunoassays, BAC is still widely used in clinical laboratories [31]. Agarose-bound *m*-aminophenylboronic acid is the most commonly used material for the enrichment of glycated amino acids [32], peptides [27, 33] or proteins [27], although optimized alternatives are available to overcome drawbacks such as high pH (> 9) and low binding capacity (< 100 µg/mL) [34, 35]. While BAC was developed for the enrichment of glucose-derived fructosamines [36, 37], some authors erroneously assume that BAC can enrich both Amadori and Heyns products equally well [38], despite discrepancies in the analysis of fructose-incubated bovine serum albumin using clinical glycation assays, including phenylboronate affinity (PBA), reported as early as 1992 [37]. These authors demonstrated the discrimination and underestimation of protein-bound HRP levels due to the lack of *cis*-diol groups in fructose-derived Heyns products, which consist of an epimeric mixture of 2-amino-2-deoxy-glucose/mannose. Due to the lack of defined standards, few studies have investigated this specificity. More recently, Morais et al. studied the interaction of differentially glycated human serum albumin (HSA) and human serum with boronic acids either by using boronic acids copolymerized in a methacrylamido phenylboronate acrylamide gel electrophoresis (mP-AGE) or by incubating samples with fluorophore-labeled boronic acids for subsequent analysis by SDS-PAGE (Flu-PAGE) and blotting (Flu-BLOT) [39, 40]. Despite a demonstrably higher rate of AGE formation, fructose and its early glycation products were characterized by both a lack of band shift [39] and comparatively low fluorescence [40], suggesting a low rate of formation or inadequate detection.

Besides HRPs, incubation with fructose can generate substantial amounts of ARPs, either by fructose isomerization [41], or by interconversion between glycation products [42]. Therefore, we investigated the enrichment efficiency of well-characterized isomeric Amadori and Heyns peptide standards by BAC using RP-HPLC in combination with targeted mass spectrometry and UV detection.

## Materials and methods

### Reagents and materials

Reagents were obtained from the following companies: AppliChem GmbH (Darmstadt, Germany): iodoacetamide (IAA); Biosolve B.V. (Valkenswaard, Netherlands): acetonitrile (ULC-MS grade, > 99.97%), formic acid (FA, > 99%, ULC-MS grade), and methanol (absolute, > 99.98%); Carl Roth GmbH (Karlsruhe, Germany): acetic acid (ROTIPURAN 100%), sodium dodecyl sulfate (SDS, ≥ 99.5%), and tris-(2-carboxyethyl)-phosphine hydrochloride (TCEP, ≥ 98%); Fluka Analytical (Seelze, Germany): ammonium bicarbonate (BioUltra, ≥ 99.5%); Sartorius AG (Göttingen, Germany): Vivaspin 2 PES concentrators (5 kDa molecular weight cut-off); SERVA Electrophoresis GmbH (Heidelberg, Germany): trypsin (NB sequencing grade, modified from porcine pancreas); Sigma-Aldrich (Steinheim, Germany): *m*-aminophenyl boronic acid-agarose (*m*APBA, aqueous suspension), ammonium acetate (≥ 98%), formic acid (~ 98%, LC–MS grade), magnesium acetate tetrahydrate (≥ 99%), sodium borohydride (≥ 98.0%), sodium hydrogen phosphate dihydrate (≥ 99.0%), and sodium dihydrogen phosphate dodecahydrate (≥ 99.0%); VWR International GmbH (Darmstadt, Germany): acetonitrile (≥ 99.9%). Water was purified in house (resistance ≥ 18 mΩ, total organic content < 1 ppb) with a PureLab Ultra Analytic System (ELGA Lab Water, Celle, Germany).

The Amadori and corresponding Heyns peptides were synthesized in-house on solid phase, with purities mostly above 90% as previously described [21, 23, 43]. The lyophilized peptides were weighed and dissolved in a 20% (v/v) aqueous acetonitrile solution to obtain a concentration of 1.5 mmol/L. Pooled human EDTA plasma was obtained from the BioMaterialBank Nord (BMB Nord) at the Forschungszentrum Borstel.

### Tryptic digest

Three aliquots of a human plasma pool (protein content 1.2 mg, determined by Bradford assay) were diluted with ammonium bicarbonate buffer (1.5 mL, 0.1 mol/L) and concentrated by ultrafiltration on Vivaspin 2 PES concentrators (5 kDa cut-off, 2880 × g, 4 °C, 25 min) to a volume of ~ 0.2 mL. Samples were diluted with ammonium bicarbonate buffer (2 mL each) and concentrated again. This procedure was repeated once, and the sample was diluted with ammonium bicarbonate buffer to a final volume of 0.5 mL. Seven aliquots of 0.4 mg protein each were diluted with solutions of SDS (20.8 µL, 0.5% in ammonium bicarbonate buffer) and TCEP (20.8 µL, 50 mmol/L in ammonium bicarbonate buffer) and incubated (60 °C, 15 min). The samples were cooled to room temperature (RT), alkylated with iodoacetamide (22.9 µL, 100 mmol/L in ammonium bicarbonate buffer) in the dark (15 min, RT), and digested with trypsin (0.8 mL, 25 mg/L in ammonium bicarbonate buffer) at 37 °C. After 16 h, samples were frozen and stored at − 80 °C.

### Boronate affinity chromatography

*m*-Aminophenylboronic acid-agarose was suspended in aqueous loading buffer (250 mmol/L ammonium acetate, 50 mmol/L magnesium acetate, pH 8.1, 4 °C), filled into empty polypropylene columns for either gravity flow (5 mL, Qiagen GmbH, Hilden, Germany) or vacuum-assisted chromatography (CHROMABOND LV columns, 15 mL, MACHEREY–NAGEL GmbH & Co. KG, Düren, Germany) to a bed volume of 1 mL and equilibrated with the loading buffer (3 × 4.5 mL). Lyophilized samples were reconstituted in 20% (v/v) acetonitrile in loading buffer (100 µL), diluted with ice-cold loading buffer (900 µL), and loaded onto the column. The flow-through (1 mL) was collected, the column was washed with loading buffer (3 × 4.5 mL; wash fraction), and glycated peptides were eluted with 0.1 mol/L acetic acid (2 × 1 mL, 2 × 3 mL) and with 0.2 mol/L acetic acid (2 mL) at 37 °C. The eluate fraction was lyophilized and, for spiked plasma digest samples, reconstituted in 200 µL of eluent B (60% (v/v) aqueous acetonitrile containing 0.1% (v/v) formic acid). The solution was diluted with 0.1% (v/v) aqueous formic acid (eluent A) to a final volume of 600 µL, transferred to polypropylene tubes (2 mL) and combined with a wash (300 µL 20% (v/v) aqueous acetonitrile containing 0.1% (v/v) formic acid) of the original/former Falcon container. Samples were frozen, lyophilized, and purified by SPE.

### Reduction

Glycated peptide standards (1.5 mmol/L in 20% (v/v) aqueous acetonitrile) were dissolved in 20% (v/v) acetonitrile in loading buffer (100 µL) to obtain a 30 µmol/L solution, which was diluted with loading buffer (375 µL). The samples were reduced by addition of a freshly prepared sodium borohydride solution (25 µL, 1 mol/L). After 1 h at room temperature, the samples were acidified with acetic acid (5 µL, 1% (v/v)) and purified by solid phase extraction (SPE). Alternatively, peptide mixtures (20 µL, 15 µmol/L in 20% (v/v) aqueous acetonitrile) were diluted in sodium phosphate buffer (14.5 mL, 50 mmol/L, pH 8.5), reduced with freshly prepared sodium borohydride solution (763 µL, 1 mol/L) under the same conditions, and acidified with acetic acid (152.6 µL, 1% (v/v)). Samples were frozen, lyophilized, and purified by SPE.

### Solid phase extraction

Samples reconstituted in eluent B (50 µL) and diluted stepwise with eluent A to a final volume of 800 µL were purified by SPE as previously reported [23]. Briefly, Oasis HLB cartridges (1 cc, 30 mg, 30 µm, Waters GmbH, Eschborn, Germany) were washed with methanol (1 mL) and equilibrated with eluent A (1 mL) for non-reduced peptides or aqueous acetic acid (0.12 mol/L, 1 mL) for reduced peptides. The sample was applied to an SPE cartridge; the stationary phase was washed with eluent A (1 mL), and the peptides were eluted in a step gradient using 333 µL of 40% (v/v), 60% (v/v), and 80% (v/v) aqueous acetonitrile containing formic acid (0.1% v/v). The combined eluate fraction was frozen and lyophilized.

### Standard solutions

Flow-through fractions (1 mL) of plasma digest samples loaded on BAC, assumed to be depleted of glycated peptides, were diluted with aqueous acetonitrile (20% v/v, 5 µL) containing either no peptide (*n* = 3) or mixtures of synthetic glycated peptides (*n* = 7) at different concentrations (Heyns: 0.25, 2.5, 25 µmol/L, Amadori: 2.5 µmol/L) and enriched with BAC as described above. Eluate fractions obtained for the “no peptide” samples were spiked with the synthetic Heyns peptide mixture (5 µL, 0.25, 2.5, 25 µmol/L) and considered as positive control samples. After lyophilization of the flow-through and elution fractions, samples were desalted by SPE, frozen, lyophilized, reconstituted in eluent B (10 µL), and diluted stepwise with eluent A to a final volume of 100 µL. For external calibration and method validation, the mixture of synthetic Heyns peptides (25 µmol/L) dissolved in aqueous acetonitrile (20% v/v) was serially diluted with aqueous acetonitrile (3% v/v) containing formic acid (0.1% v/v) to obtain concentrations ranging from 2 µmol/L to 2 pmol/L. Samples were analyzed by RP-HPLC-ESI-QqLIT-MS in multiple reaction monitoring (MRM) mode. Peptide recoveries were determined by comparing peak areas of transitions for either fructose-specific neutral losses (qualifier) or unglycated peptide backbone fragment ions (quantifier) between enriched and corresponding non-enriched samples.

Alternatively, Heyns and Amadori peptides were used in two complementary mixtures, each containing one of the two isomeric peptides, avoiding the difficult differentiation of the isomers while representing both modifications in each sample (Tab. S5). The glycated peptides or the corresponding reduced versions were diluted or dissolved in acetonitrile (20% v/v) in loading buffer to concentrations of 30 µmol/L or 60 µmol/L, diluted fivefold with ice-cold loading buffer, applied to BAC, and eluted as described above. Fractions were lyophilized, dissolved in eluent B (50 µL), diluted stepwise with eluent A to a final volume of 800 µL, and desalted by SPE unless otherwise noted. Samples were reconstituted in eluent B (10 µL), diluted stepwise with eluent A to a final volume of 120 µL, and analyzed by RP-HPLC-ESI-IT-MS. Peptide recoveries were determined by comparing the peak areas obtained for the original standard peptide mixture and the BAC fractions by recording the absorbance at 214 nm.

### RP-HPLC-ESI-IT-MS

Samples (100 µL) were separated on a Jupiter C_18_ column (ID: 2 mm, length: 150 mm, particle size: 5 µm, pore size: 300 Å, Phenomenex, Aschaffenburg, Germany) using an 1100 LC system (Agilent Technologies, Böblingen, Germany) with a linear gradient from 95% eluent A to 95% eluent B in 30 min. Alternatively, samples were analyzed on an Aqua C_18_ column (ID: 2 mm, length: 150 mm, particle size: 3 µm, pore size: 125 Å, Phenomenex) using an ACQUITY Arc system (Waters GmbH) equipped with a 2489 UV/VIS detector. Separations were based on a linear gradient from 97% eluent A to 40% eluent C (acetonitrile with formic acid (0.1% v/v)) in 37 min. Separations were performed on both columns at a temperature of 60 °C and a flow rate of 0.2 mL/min. The absorbance was recorded at 214 nm. Both HPLC systems were coupled online to an Esquire HCT ion trap mass spectrometer equipped with an electrospray ionization source (ESI-IT-MS, Bruker Daltonics). The ESI source was operated in positive ion mode at a source temperature of 365 °C using nitrogen as curtain gas (40 psi) and dry gas (9 L/min).

### RP-HPLC-ESI-QqLIT-MS (MRM)

Heyns peptides were diluted in eluent B (0.5 µmol/L, #1:0.75 µmol/L) and infused at a flow rate of 5 µL/min (PHD 2000 Infusion Syringe Pump, Harvard Apparatus, Holliston, USA) into an ESI-QqLIT-MS (4000 QTRAP, AB Sciex, Darmstadt, Germany) equipped with a Turbo V ion source operating in positive ion mode (Tab. S1). The settings for the declustering potential (DP), collision potential (CE), and collision cell exit potential (CXP) were adjusted using the Analyst® 1.6 software (AB Sciex) to obtain an optimal signal response for the characteristic neutral loss of 96 Da, which is considered specific for ketohexose-derived compounds (Tab. S2). Processed samples (80 µL) or calibration peptide mixtures (50 µL) were analyzed by RP-HPLC on an AdvanceBio Peptide Mapping C_18_ column (ID: 2.1 mm, length: 150 mm, particle size: 2.7 µm, pore size: 120 Å, Agilent Technologies) using an Alliance® 2695 HPLC system (Waters GmbH) coupled online to the 4000 QTRAP. Peptides were separated under isocratic conditions (97% eluent A) for 3 min, followed by linear gradients to 10% eluent C in 1 min, to 20% eluent C in 10 min, and to 95% eluent C in 7 min, using a column temperature of 60 °C and a flow rate of 0.3 mL/min. Data were acquired based on a previously reported scheduled MRM method [44], adjusted for Heyns-specific transitions of seven glycated peptides (Tab. S2). Peak integration of MRM transitions was performed in the extracted ion chromatograms (XICs) using Analyst 1.6 software (AB Sciex).

### ESI-QqOrbitrap-MS (PRM)

Peptide standards were diluted in either eluent B or a mixture of acetonitrile (50% v/v) and methanol (25% v/v) in water containing acetic acid (1% v/v) to a final concentration of 1 µmol/L and infused with a syringe pump (model: Fusion 100 T, Chemyx Inc., Stafford, TX, USA) at flow rates of 25 to 50 µL/min into a Q Exactive Plus Hybrid Quadrupole-Orbitrap MS (Thermo Fisher Scientific) equipped with a HESI probe operating in positive ion mode. Instrument parameters were tuned for the highest signal intensity representing doubly and triply charged precursor ions around *m*/*z* 1000: spray voltage of 3.5 kV, capillary temperature of 300 °C, heater temperature of 370 °C, S-lens RF value of 60, sweep gas flow of 1 L/min, sheath gas of 40 psi, and auxiliary gas of 10 psi. The acquisition method was based on a survey MS scan followed by up to two targeted MS/MS scans defined by a scheduled (0.1–0.25 min) inclusion list selecting two precursors per analyte (Tab. S3). Mass spectra were acquired in a scan range of *m*/*z* 400 to *m*/*z* 1500 with a resolution of 70,000 at *m*/*z* 200, a maximum IT of 240 ms, and an AGC target of 10^6^ ions. Targeted tandem mass spectra were acquired in an automatically defined scan range (“fixed first mass” disabled) with a resolution of 35,000 at *m*/*z* 200, a maximum IT of 100 ms, and an AGC target of 2 × 10^5^ ions. Precursor ions were selected with an isolation window of 0.7 *m*/*z*-units and fragmented by HCD using individually optimized NCEs (Tab. S3).

## Results and discussion

The enrichment of low abundant Amadori peptides by boronate affinity chromatography is a prerequisite for their reliable quantitation in plasma samples by targeted bottom-up proteomics [24], as coeluting highly abundant peptides and other plasma components would otherwise significantly suppress their signals and reduce the linear dynamic range (matrix effects). Similarly, we anticipated that the analysis of Heyns peptides, presumably present at even lower concentrations than Amadori peptides, would require affinity enrichment of plasma sample digests to allow quantitation by a recently established targeted PRM assay [21]. The previously identified Heyns- and Amadori-specific fragmentation patterns, which allowed the quantitation of coeluting Amadori and Heyns peptide isomers in digests of in vitro glycated recombinant HSA (rHSA), detected only the Amadori peptides but not the Heyns peptides in enriched plasma samples. The Heyns peptides were also not detected in the flow-through of the BAC, which could be due to either high sample complexity and low levels of Heyns peptides in the eluate, or strong/unspecific binding to the stationary phase or the solid support. Therefore, this study investigated the enrichment of Amadori and Heyns peptides in BAC.

### Quantitation of glycated peptide isomers in BAC-enriched fractions

Reliable quantitation of Heyns peptides in human plasma digests was based on synthetic peptide standards infused into a triple quadrupole mass spectrometer to obtain optimized settings for very intense neutral losses of 96 Da, specific for fructose-derived Heyns products. These transitions, based on characteristic losses of predominantly doubly and triply protonated precursor ions (Fig. S1), were implemented in a MRM method previously reported for the corresponding Amadori peptides [23], consisting of three transitions per peptide (Tab. S2). Similar to a recent PRM assay [21], transitions based on non-glycated backbone fragment ions provided unbiased quantitation of both glycation isomers (quantifier), while the 96 Da loss predominantly reflected the Heyns modification (qualifier). When serially diluting a mixture of Heyns peptides in aqueous buffer, these qualifier transitions showed equally good linearity (Fig. S2a/b) with the LOQ typically two to five times higher than the LOD (Tab. S4). This is mainly due to a lower signal intensity observed for the lower charged quasimolecular ions selected for quantitation (precursor neutral loss).

**Fig. 1:**
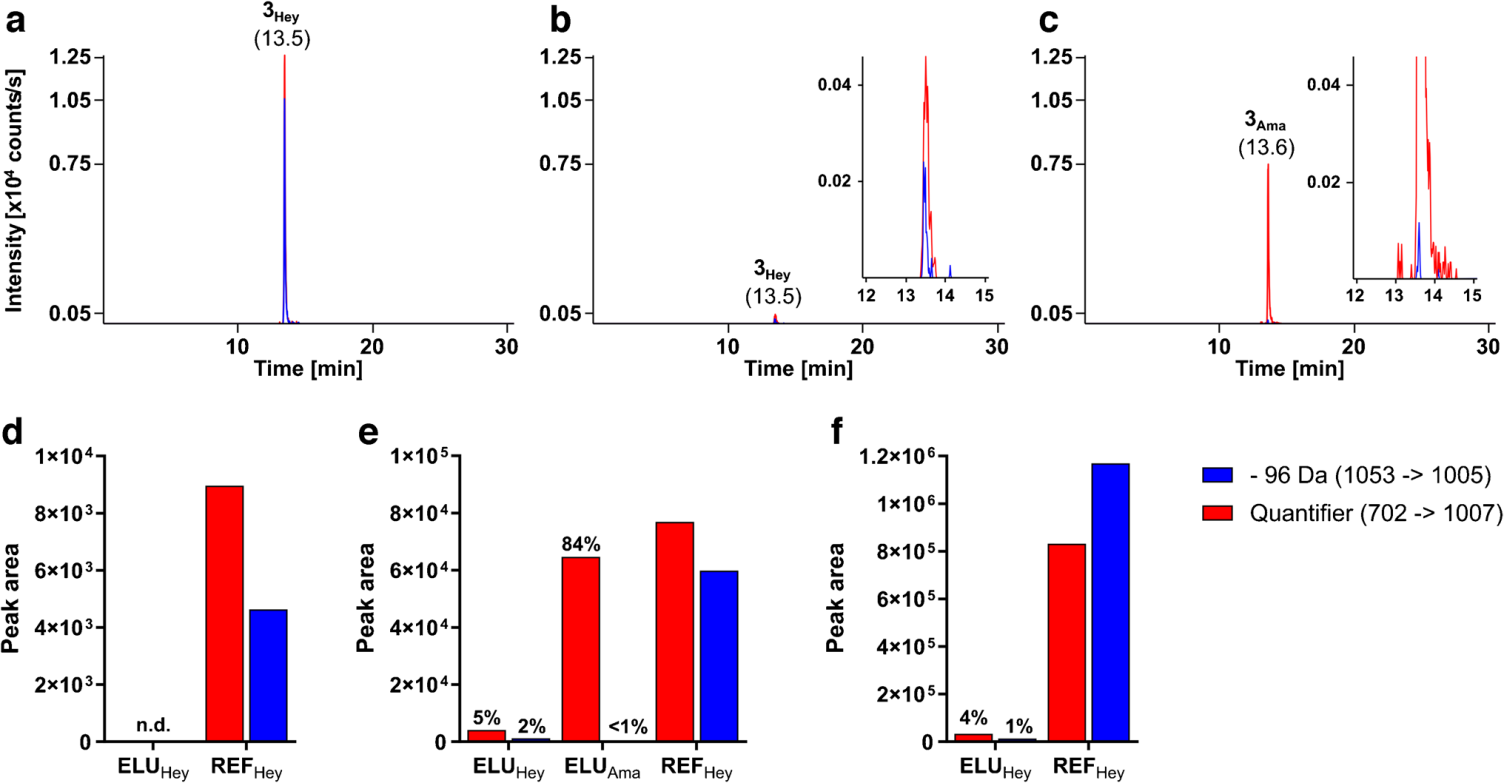
Overlays of XICs (**a**–**c**) and peak areas (normalized based on UV absorbance in RPC, **d**–**f**) of glycated peptide #3 (ADLAK_Hex_YIC_CAM_ENQDSISSK) after affinity enrichment (ELU, **b**, **c**) and without enrichment (REF, **a**) of a glycation-depleted plasma matrix spiked with either synthetic Amadori (**c**, **e**) or Heyns peptides (**a**, **b**, **d**–**f**) at concentrations of 1.25 pmol (**d**), 12.5 pmol (**a**–**c**, **e**), and 125 pmol (**f**). The XICs of the doubly protonated precursor ion are shown for the MRM transitions *m*/*z* 1053 → 1005 (Heyns qualifier, blue) and *m*/*z* 702 → 1007 (quantifier, red) with 80% of the sample injected into the LC–MS system (on-column). Recovery rates in elution fractions are summarized in Tab. S5 (Exp 1/Exp 2)

The flow-through of a pooled human plasma digest loaded onto the BAC column was divided into seven equal aliquots. Three aliquots were spiked with different quantities of a mixture of eight Heyns peptides (1.25 pmol, 12.5 pmol, or 125 pmol) to investigate their retention in the BAC for complex plasma digests. An additional aliquot was spiked with the corresponding Amadori peptides (12.5 pmol) to confirm their binding to the same agarose affinity resin under the same conditions. These four samples and the three unspiked matrix samples were applied to BAC columns. The eluate fractions of the unspiked samples were spiked with the Heyns peptide mixture at different quantities (1.25 pmol, 12.5 pmol, or 125 pmol, reference control). All eluate fractions were dried, desalted by SPE, and analyzed in MRM mode using on-column loads of 1 pmol, 10 pmol, or 100 pmol per peptide (80 µL injection volume). As all Amadori and all Heyns peptides showed very similar binding and elution behavior on BAC, we will describe the results for the representative peptide #3 (ADLAK_Hex_YIC_CAM_ENQDSISSK). The XICs of the mass spectra recorded in MRM mode for initial peptide quantities of 12.5 pmol displayed the signals of the quantifier (red, transition 702 → 1007) at high intensities (Fig. 1a).

**Fig. 2:**
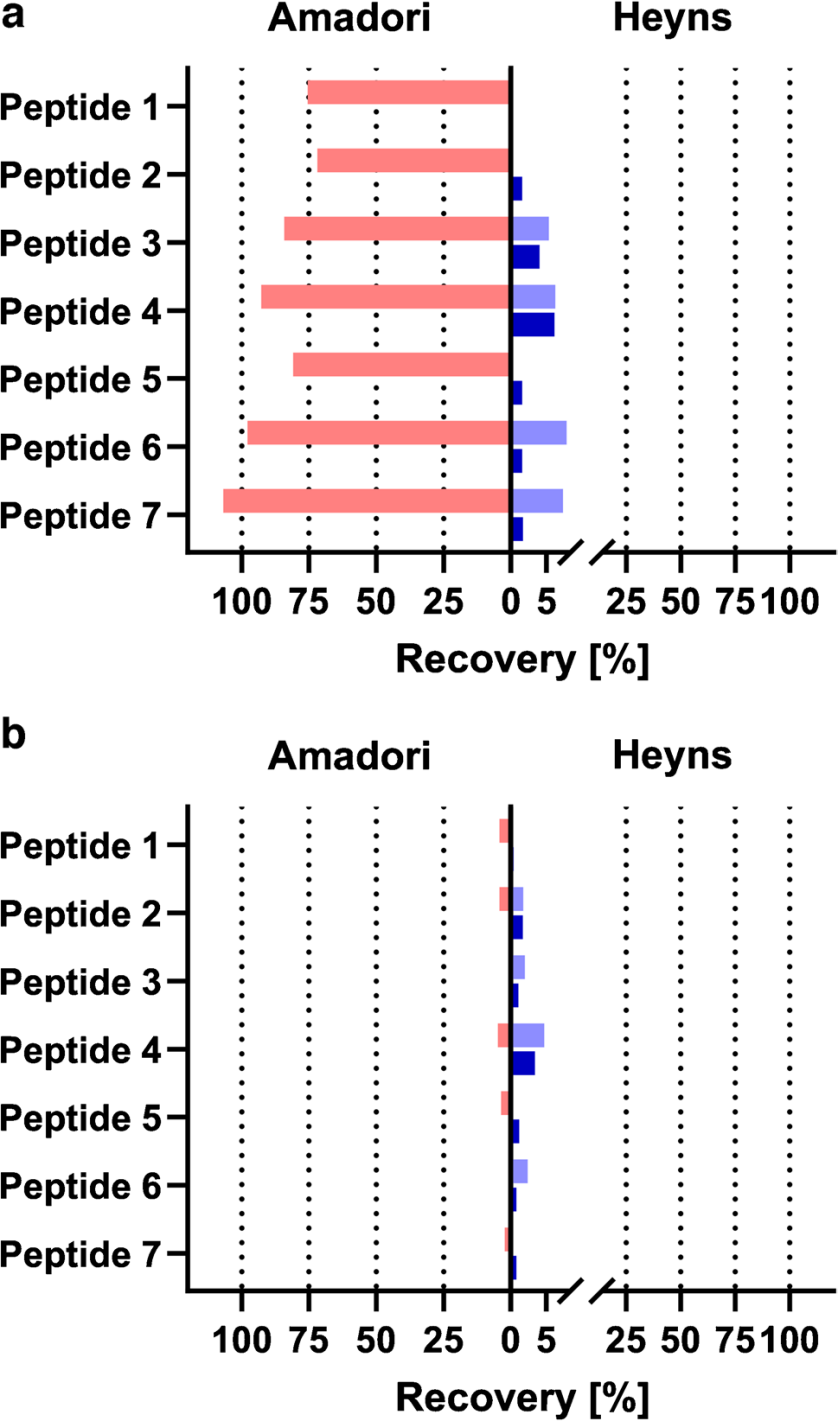
Recovery rates of seven different Amadori (12.5 pmol, red) and Heyns peptides (12.5 pmol, light blue; 125 pmol, dark blue) in affinity-enriched fractions calculated from peak areas of quantifier (**a**, non-glycated backbone fragment ion) or qualifier (**b**, fructose-characteristic neutral loss of 96 Da) MRM transitions. Peptide sequences and modification sites are listed in Tab. S3. Recovery rates in elution fractions are summarized in Tab. S5 (Exp 1/Exp 2)

Expectedly, both quantifier and Heyns qualifier transitions (blue, transition 1053 → 1005) showed identical chromatographic profiles with a retention time of 13.5 min. The identical retention times were observed for the Amadori and Heyns peptides after BAC enrichment (Fig. 1b, c). The Amadori peptide was detected with a quantifier peak area corresponding to 84% of the quantity of peptide loaded on BAC (Fig. 1c). In contrast, the Heyns peptide showed a very weak quantifier signal of only 5%, and its qualifier signal (96 Da loss) was close to the detection limit (Fig. 1b). The qualifier signal of the Heyns peptide was also observed for the Amadori peptide at a very low intensity, which was similar to the signal intensity observed for the enriched Heyns peptide (zoomed insets in Fig. 1b, c). Although the ratio of quantifier to (false) qualifier peak area was much lower than for Heyns peptides, it highlights the risk of misleading annotation of Amadori peptides as Heyns peptides present at low levels. The reference controls (REFs) containing 1.25, 12.5, or 125 pmol Heyns peptide (80% injected on-column, i.e., theoretical injections on-column of 1, 10, or 100 pmol, respectively) displayed intense signals of the quantifier (non-glycated y_9_^+^) that increased linearly with concentration. The HRP-characteristic neutral loss of 96 Da (qualifier) showed a different characteristic, as its peak area increased relative to the quantifier from ~ 50% at 1 pmol on-column to 125% at 100 pmol on-column (Fig. 1a, d–f, Fig. S2c-f). Peptide losses during sample preparation reduced the signal intensities of the neutral loss qualifier transitions for 10 pmol loading to the lower linear dynamic range (LLDR) of 5 pmol (Tab. S4). A peptide load of 1.25 pmol in the reference controls still allowed detection of both transitions representative of the Heyns peptides, while these signals were not detected after affinity enrichment of the same peptide load (Fig. 1d). The affinity-enriched fractions obtained by loading 12.5 pmol (Fig. 1e) and 125 pmol (Fig. 1f) showed weak quantifier signals sufficient for reliable quantitation, while the qualifier was detected only for the higher load. The XICs indicated recovery rates equal to or less than 5% for both peptide loads, confirming the weak binding of fructose-derived Heyns peptides in BAC, at least for the standard conditions applied here. In contrast, the quantifier peak area of the enriched Amadori peptide showed an efficient recovery rate of 84%, confirming the high affinity of BAC for the glucose-derived isoform (Fig. 1e). The other six peptide sequences supported the strong discrimination of Heyns peptides on BAC, while the enrichment of Amadori was generally effective, with hydrophobic peptides (peptide #6 and #7) showing slightly higher recovery rates (Fig. 2a, Tab. S5 Exp 1/2).

Amadori peptides were enriched with an average recovery of 87%, but not less than 72% (peptide #2). The quantifier (and qualifier) signals of all Heyns peptides were intense enough to determine recovery rates at 125 pmol loading, ranging from 0.3% (0.4%) for peptide #1 to 6.1% (3.4%) for peptide #4, with a mean recovery of 2%. At the 12.5 pmol scale, four peptides could be quantified with average recovery rates of 7% and 3% based on the quantifier and qualifier signals, respectively, while the other peptides showed signals below the LOQ corresponding to a recovery rate of 1%.

Given the low enrichment rates by BAC, the Heyns peptides are expected to be present in the flow-through or wash fractions. However, the quantifiers and qualifiers were detected with very low signal intensities in these fractions, even for the highest peptide loads, which could not be reliably quantified (data not shown). Since the high sample complexity of the flow-through may suppress the peptide signals, BAC binding was also tested in the absence of a digestion matrix.

**Fig. 3:**
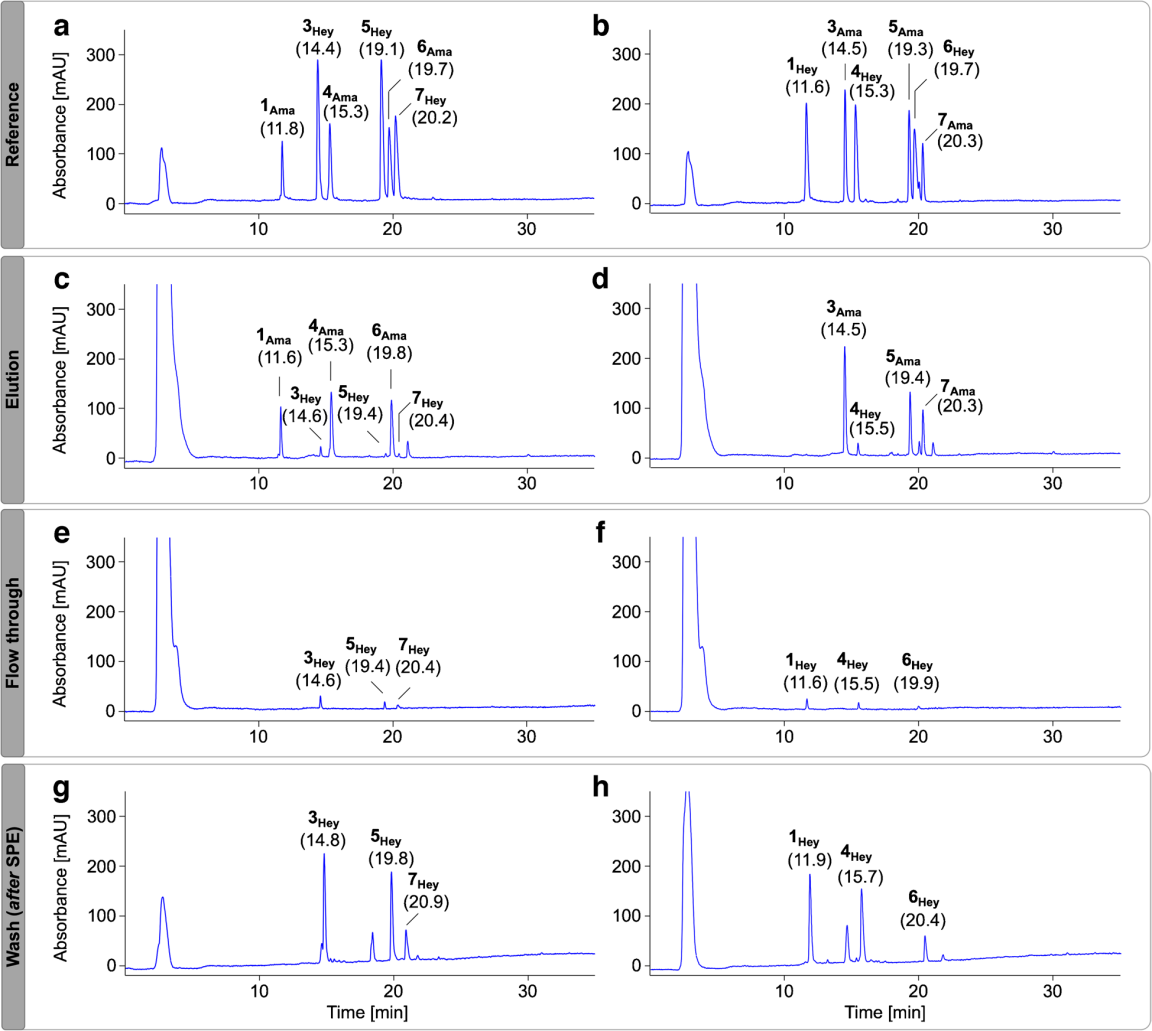
RP chromatograms of two complementary peptide mixtures (**a**, **b**) containing three fructated (Hey) and three glucated peptides (Ama, 1 nmol each) and the corresponding elution (**c**, **d**), flow-through (**e**, **f**), and SPE-purified wash fractions (**g**, **h**) collected by BAC. Ammonium acetate loading buffer (250 mmol/L, 50 mmol/L magnesium acetate, pH 8.1) was used for equilibration, sample loading, and washing of the BAC column. Peptides were separated on a Jupiter C_18_ column at 60 °C using a linear gradient from 95% eluent A to 95% eluent B in 30 min. The absorbance was recorded at 214 nm. Peptide sequences and modification sites are listed in Tab. S3. Recovery rates and percentage distribution in affinity processed fractions are summarized in Tab. S5 and Tab. S6 (Exp 3)

Therefore, a mixture of three Heyns and three Amadori peptides as well as a second mixture containing the corresponding isomeric three Amadori and three Heyns peptides (peptide #1 was not included) was loaded onto BAC and the flow-through, wash, and elution fractions were separated by RP-HPLC coupled on-line to a UV detector (absorbance recorded at 214 nm) and an ESI ion trap (IT) mass spectrometer. The analysis of two isomeric peptide mixtures avoided the coelution of isomeric glycated peptides in RP-HPLC and allowed their specific detection, while loading both peptide modifications in each sample onto BAC. The lower sensitivity of UV detection required higher peptide loads in the low nanomolar range (6 nmol per peptide). As expected, recoveries of Amadori peptides in the elution fractions ranged from 58% (peptide #5) to 89% (peptide #3) (Fig. 3c, d), while the maximum recovery of Heyns peptides was 6% (peptide #4). Similar quantities of the Heyns peptides were also detected in the flow-through (Fig. 3e, f, Tab. S5/S6 Exp 3). The high salt content of the wash fractions required desalting by SPE (Fig. S3). Although this may result in partial peptide loss, 23% (peptide #6) to 87% (peptide #1) of the Heyns peptides were present in the wash fraction. The additional drying and reconstitution steps used for BAC, as well as poor elution from the SPE, may explain the lower recoveries of hydrophobic peptides, especially peptide #6. These data suggest that Amadori peptides can be enriched by BAC with high recovery rates, while the high proportion of Heyns peptides in the wash fraction suggests their very weak binding.

### Effect of loading buffer on the enrichment of glycated peptides

So far, samples for BAC were reconstituted and/or diluted with loading buffer (250 mmol/L ammonium acetate, 50 mmol/L magnesium acetate, pH 8.1), which was also used for subsequent washing. In order to improve Heyns peptide retention by favoring boronate ester formation, a higher pH during column equilibration (pH 10) and a lower salt content (50 mmol/L phosphate buffer, pH 8.5) in the loading buffer were tested on a new batch of *m*-aminophenylboronic acid-agarose using the above-mentioned peptide mixtures (3 nmol per peptide). The flow-through and wash fractions of BAC and the reference standard peptide mixture dissolved in acetic acid (0.12 mol/L) were dried and desalted by SPE.

**Fig. 4:**
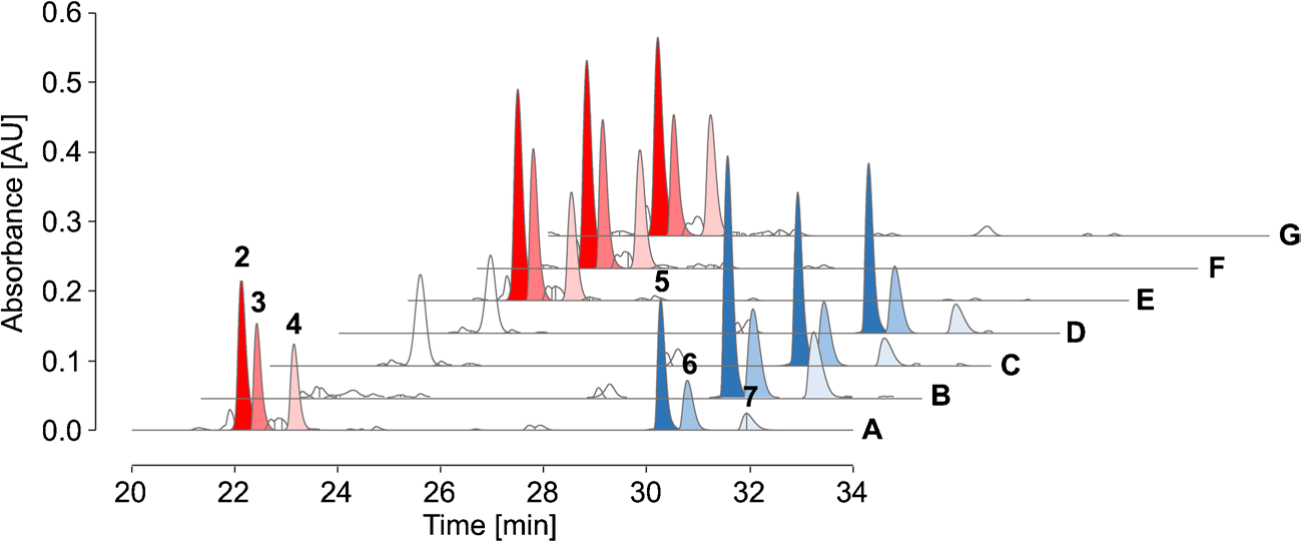
RP chromatograms from 20 to 34 min of an SPE-purified peptide mixture (A) containing three fructated (Heyns, blue peaks) and three glucated peptides (Amadori, red peaks, 0.75 nmol each) as well as unbound (B–D) and enriched fractions (E–G) collected by BAC. Equilibration, sample loading, and column washing in BAC were performed using either sodium phosphate (50 mmol/L, pH 8.5, B/E) or ammonium acetate loading buffer (250 mmol/L, 50 mmol/L magnesium acetate, pH 8.1 C/F), the latter also with additional equilibration at pH 10 (D/G). Peptides were separated on an Aqua C_18_ column at 60 °C using a linear gradient from 97% eluent A to 40% eluent C in 37 min. The absorbance was recorded at 214 nm. Peptide sequences and modification sites are listed in Tab. S3. Results for the complementary peptide mixture are shown in Fig. S4. Recovery rates and percentage distribution in affinity processed fractions are summarized in Tab. S5 and Tab. S6 (Exp 4/Exp 5/Exp 6)

Amadori peptides were equally well enriched in the elution fractions for all three conditions (Fig. 4E–G), whereas none of the conditions improved binding of Heyns peptides, as indicated by high signal intensities in the combined flow-through and wash fractions (Fig. 4B–D, Tab. S5/S6 Exp 4–6). Control samples, treated similarly to the eluate fraction, showed slightly lower peak areas than the BAC fractions (Fig. 4A), indicating that salt remaining from the loading buffer may improve recovery rates in SPE.

Phenylboronate ester formation is strongly favored by *syn*-periplanar sugar hydroxyl groups, which are preferentially present in their furanosyl tautomeric form, explaining significant binding differences among monosaccharides (fructose > galactose > mannose > glucose) in BAC [22, 26]. Under aqueous conditions, the relatively high fructofuranosyl content of ARPs enables strong binding of anomeric *cis*−1,2-diols as boronate esters, which is further stabilized by electrostatic interactions between the protonated amino group and the negatively charged boronate anion [45–47]. Excess phenylboronate ions also allow 2,3- and the 4,5-*cis*-diol groups of the β-pyranose anomer to be captured as a bisboronate species [45], which can occur in BAC when phenylboronate groups are in close proximity. This is in strong contrast to the epimeric N-substituted 2-amino-2-deoxy-glucose and -mannose forms of fructose-derived Heyns peptides, which preferentially adopt a gluco/mannopyranosyl form without *cis*-diols and only to a small extent the mannofuranosyl form with *cis*-diols under aqueous conditions [20, 43, 48, 49]. This supports the results that Heyns peptides bind very weakly in BAC, which is further supported by structurally related glucose-derived Schiff bases, labile hydroxyaldimines, which cyclize in aqueous solutions to N-substituted aldosylamines [45, 49].

### Boronate affinity chromatography of reduced glycated peptides

McPherson et al. reported the identification of protein-bound Heyns rearrangement products after reduction in vitro and in vivo in the human ocular lens using very similar affinity conditions at the amino acid level in protein hydrolysates [50]. The intention of reducing the carbonyl group to a hydroxyl group with sodium borohydride prior to acid hydrolysis was to obtain a stable carbohydrate-protein linkage. The reduction is important for Amadori products, as they would otherwise hydrolyze completely within 18 h, while Heyns products are stable for even longer periods [16, 19]. The 1-deoxy-1-amino-hexitols obtained from Amadori products are linear and thus have a high affinity for boronic acids, such as N-methyl-d-glucamine, which is a model compound for reduced ARP [50]. Similarly, the 2-amino-2-deoxy-hexitols obtained from Heyns products have the potential for stronger interactions in BAC due to a more favorable hydroxyl group orientation. This was observed for free aldoses, which showed 4- to 25-fold increased binding affinities to boronic acid receptors upon reduction [51].

**Fig. 5:**
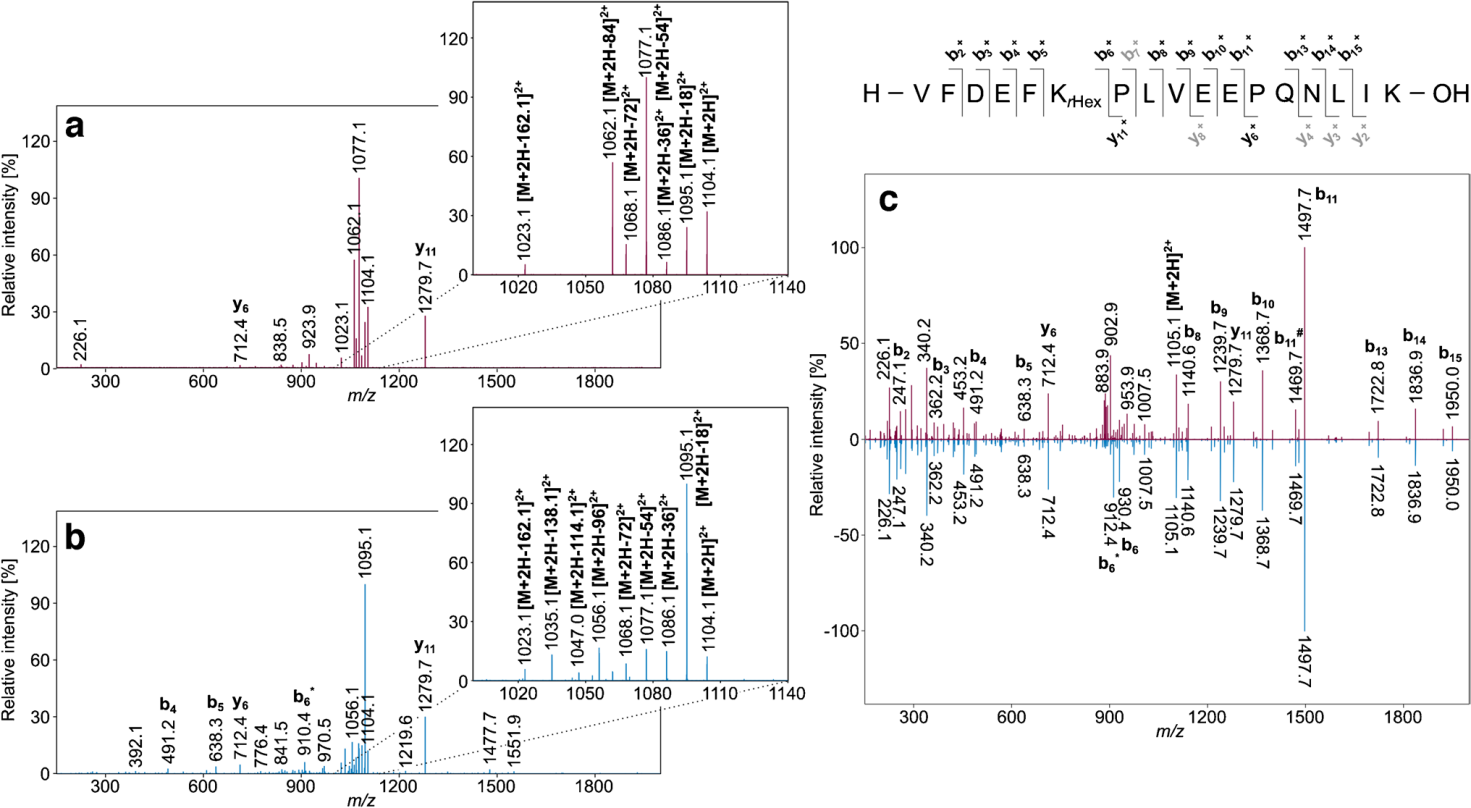
Tandem mass spectra of doubly protonated precursor ions of Amadori (red) or Heyns (blue) modified peptide #5 (VFDEFK_Hex_PLVEEPQNLIK, **a**, **b**, *m*/*z* 1104.1) and the corresponding reduced peptides (**c**, *m/z* 1105.1) using normalized collision energies (NCE) of 22% and 30%, respectively. Signals indicating neutral losses of water (H_2_O) and carbon monoxide (CO) are marked with asterisks (*) and hashtags (#), respectively. Gray colored b- and y-fragment ions were detected, but they are not annotated in the tandem mass spectra. The insets show enlarged mass ranges including the characteristic neutral loss pattern of unreduced glycated peptides. Raw and annotated tandem mass spectra of all glycated, reduced glycated, and unmodified peptides are provided in the supplement (Fig. S9-S20)

This could allow a strategy to specifically enrich both Amadori and Heyns peptides by using BAC to enrich Amadori peptides, followed by a second BAC of the reduced flow-through to enrich Heyns peptides as their corresponding hexitol derivatives. First, the two complementary glycated peptide standards were reduced with sodium borohydride in either phosphate buffer or ammonium acetate loading buffer for 1 h and desalted by SPE. The glycated and the corresponding reduced glycated peptides, i.e., (1-deoxy-glucitol/mannitol-1-yl)-lysine and (2-deoxy-glucitol/mannitol-2-yl)-lysine derivatives, coeluted exactly and the peak areas did not change when the absorbance was recorded at 214 nm. However, the peptide masses increased by ~ 2 Da (Fig. S5). The relative signal intensities of the monoisotopic masses of doubly and triply protonated molecular ions of the reduced (+ 164.0685 Da, C_6_H_12_O_5_) and unreduced glycated peptide isoforms (+ 162.0528 Da, C_6_H_10_O_5_) indicated an almost quantitative reduction of both Amadori and Heyns peptides when the reaction was performed in phosphate buffer (Fig. S6). While glycated peptide isomers show characteristic and intense neutral loss patterns in collision-induced dissociation (CID) (Fig. 5a, b), none of the reduced Amadori or Heyns peptides showed fragmentation of hexitol at the same collision energy (Fig. S7c). Higher collision energies induced the same fragmentation patterns in both reduced Amadori and Heyns peptides; multiple water losses leading to oxonium, pyrylium, or furylium ions were not observed (Fig. 5c, Fig. S9-20). The spectra displayed dominant peptide backbone fragmentation with several high intense y- and b-ion signals, including the modified lysine residue. This tandem mass spectra looked similar to mass spectra recorded by electron transfer dissociation (ETD) reported for Amadori peptides [52]. Although this simplifies peptide sequencing for both Amadori and Heyns peptides, as has been repeatedly shown for glycated protein digests [53, 54], the data do not allow Amadori and Heyns peptides to be distinguished.

**Fig. 6:**
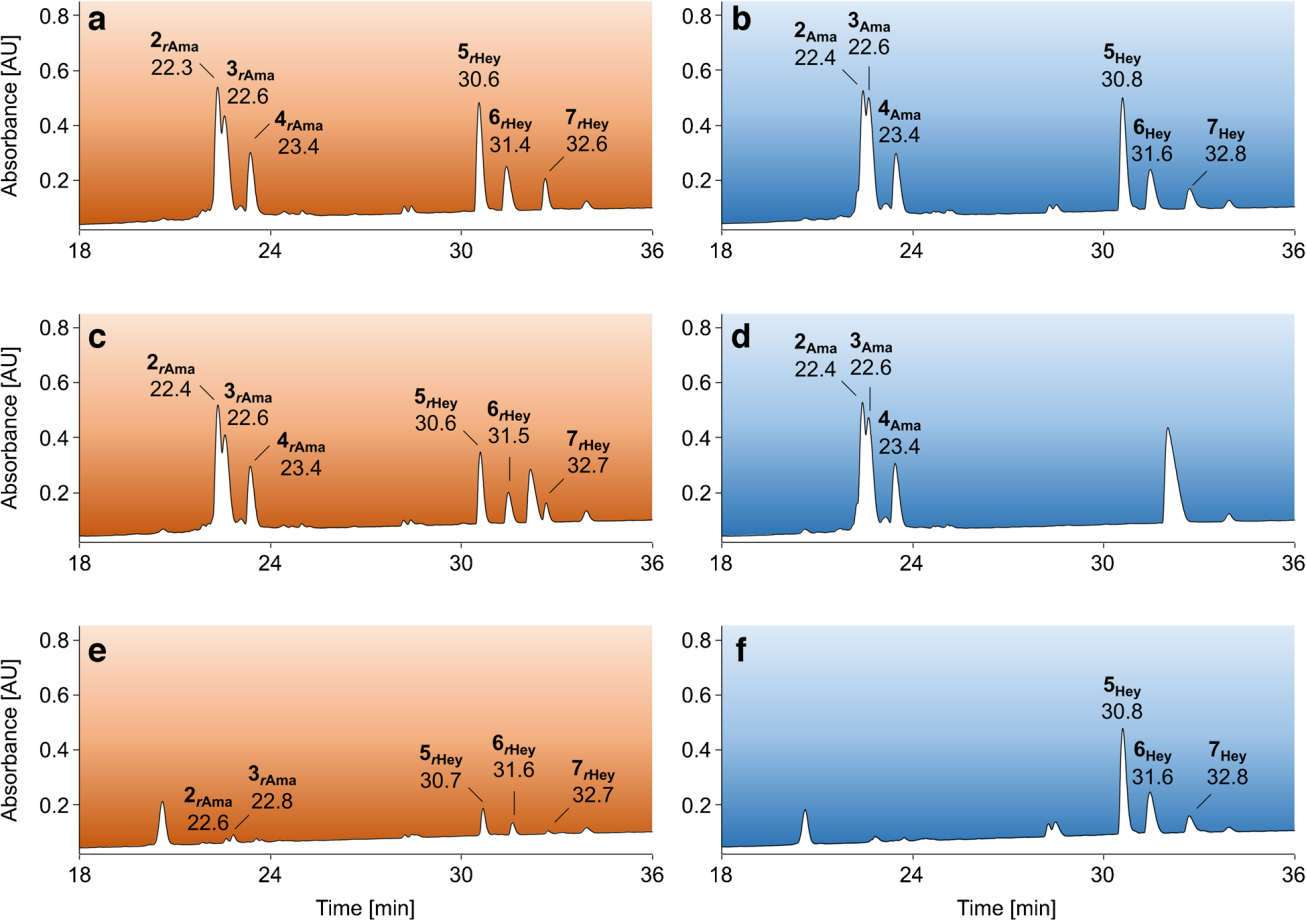
RP chromatograms from 18 to 36 min of an SPE-purified peptide mixture containing three fructated (Hey) and three glucated peptides (Ama, 1 nmol each) incubated in sodium phosphate buffer (50 mmol/L, pH 8.5) in the presence (orange, *r*Ama/*r*Hey, **a**) or absence (blue, **b**) of sodium borohydride and the corresponding elution (**c**, **d**) and wash fractions (**e**, **f**) obtained by BAC using ammonium acetate loading buffer (250 mmol/L, 50 mmol/L magnesium acetate, pH 8.1). Peptides were separated on an Aqua C_18_ column at 60 °C using a linear gradient from 97% eluent A to 40% eluent C in 37 min (absorbance recorded at 214 nm). Peptide sequences and modification sites are listed in Tab. S3. Results for the complementary peptide mixture are shown in Fig. S21. Recovery rates and percentage distribution in affinity processed fractions are summarized in Tab. S5 and Tab. S6 (Exp 7)

Second, the two complementary peptide mixtures were incubated with or without sodium borohydride in phosphate buffer, desalted, and loaded onto BAC in ammonium acetate loading buffer. Expectedly, without reduction, the Amadori peptides were detected in the eluate fraction (Fig. 6d) and the Heyns peptides in the flow-through (Fig. 6f). In contrast, all reduced peptides were very well enriched (Fig. 6c), although the enrichment for hydrophilic sequences was more efficient than for hydrophobic sequences using retention times as a measure of polarity. Early eluting reduced Heyns peptides #2, #3, and #4 were enriched with recovery rates of 86%, 77% and 90%, respectively (see complementary mixture in Fig. S21), while later eluting peptides #5, #6, and #7 were enriched with recovery rates of 56–59% (Fig. 6c, Tab. S5/S6 Exp 7). Similar results were obtained for corresponding reduced Amadori peptides, i.e., 93 to 95% (Fig. 6c) and 41 to 57% (Fig. S21), respectively.

**Fig. 7:**
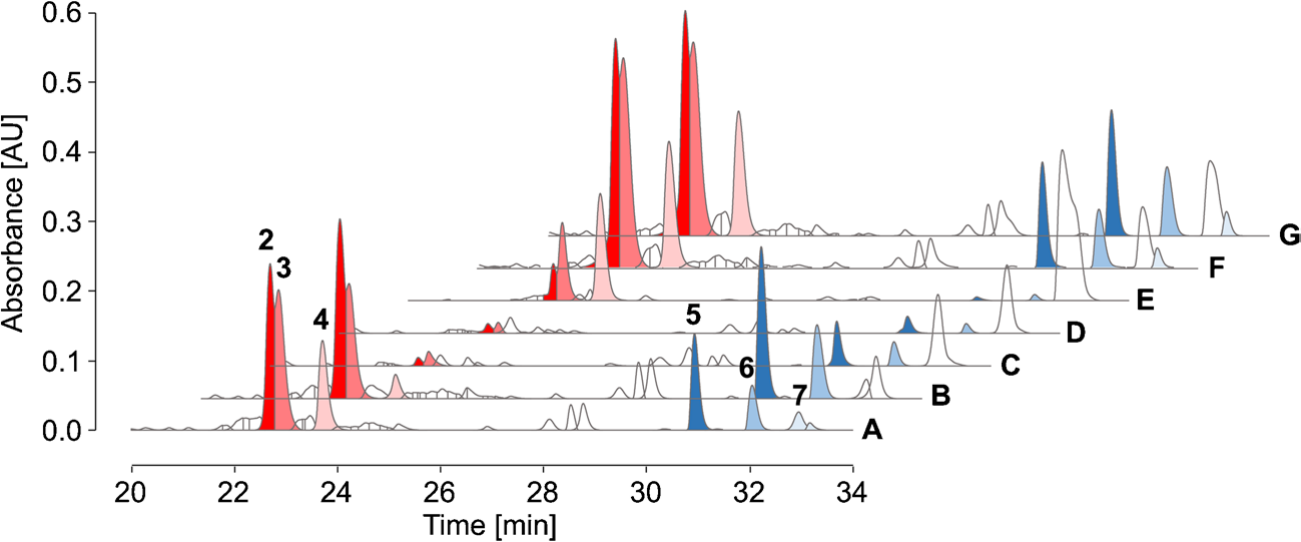
RP chromatograms from 20 to 34 min of an SPE-purified peptide mixture (A) containing three fructated (Heyns, blue peaks) and three glucated peptides (Amadori, red peaks, 1 nmol each) reduced with sodium borohydride and the unbound (B–D) and enriched fractions (E–G) collected by BAC. Equilibration, sample loading, and column washing in BAC were performed using either sodium phosphate (50 mmol/L, pH 8.5, B, E) or ammonium acetate loading buffer (250 mmol/L, 50 mmol/L magnesium acetate, pH 8.1 C, F), the latter also with additional equilibration and sample loading at pH 10 (D/G). Peptides were separated on an Aqua C_18_ column at 60 °C using a linear gradient from 97% eluent A to 40% eluent C in 37 min. The absorbance was recorded at 214 nm. Peptide sequences and modification sites are listed in Tab. S3. Results for the complementary peptide mixture are shown in Fig. S22. Recovery rates and percentage distribution in affinity processed fractions are summarized in Tab. S5 and Tab. S6 (Exp 8/Exp 9/Exp 10)

Third, the complementary peptide mixtures were reduced with the same borohydride concentration (50 mmol/L) in phosphate loading buffer, but using a larger volume (14.5 mL) to closely simulate the composition of the flow-through fraction in BAC. While very similar recovery rates were obtained with ammonium acetate loading buffer (pH 8.1), phosphate loading buffer (pH 8.5) provided much poorer enrichment with a large proportion of the peptides present in the flow-through (Fig. 7), except for peptide #4 (Fig. 7E, Tab. S5/S6 Exp 8–10). This difference could be explained by better binding of the N-terminally glycated lysine residues compared to midchain positions or the presence of adjacent serine and threonine residues that could form stable boronates. When the column was equilibrated and the samples were loaded in ammonium acetate loading buffer (pH 10), the recovery rates improved further, as more alkaline conditions should favor boronate formation. However, this required additional rebuffering after reduction in phosphate buffer.

## Conclusion

While boronate affinity chromatography was originally described for the enrichment of 1,2- and 1,3-*cis*-diols, such as glucose-derived fructosamines (Amadori peptides), it has also been used in recent literature to enrich fructose-derived glucosamines (Heyns peptides). Here, we could confirm that BAC with phenylboronic acid specifically enriches Amadori peptides, while Heyns peptides bind only weakly or not at all in alkaline phosphate or ammonium acetate buffers. After reduction with sodium borohydride, the resulting 1- or 2-hexitol-lysine derivatives were enriched in BAC with moderate to excellent recovery rates from ~ 50% to nearly 95%, with hydrophobic sequences in the lower range. Tandem mass spectra of reduced glycated peptides recorded in CID mode showed dominant peptide backbone fragmentation, ideal for peptide sequencing, but the absence of indicative neutral losses prevented differentiation of isomeric glycation products. Our results clearly show that BAC-based assays are specific for Amadori products and almost quantitatively discriminate Heyns products. The contribution of fructose to the formation of early glycation products in vivo has likely been underestimated in the literature. This might also be true for glycation products formed at the N-terminus of proteins, such as HbA_1c_, although further research is needed to confirm this. Future studies have to show whether the established method is able to analyze fructose-induced glycation in protein mixtures in vitro and, more importantly, in complex in vivo samples, such as plasma.

## Supplementary Information

Below is the link to the electronic supplementary material.

ESM 1 Supplementary Material 1: LC–MS settings; optimized collision energies and source parameters in scheduled MRM; sensitivity and linearity parameters for glycated peptides; XICs of qualifier and quantifier transitions for Heyns peptide spiked plasma samples depleted of Amadori peptides; precursor list and optimized normalized collision energies in direct infusion MS/MS (PRM); recovery rates and percentage distribution of glycated and reduced glycated peptide standards in affinity fractions; raw and annotated tandem mass spectra of unmodified, glycated and reduced glycated peptide standards; RP chromatograms of affinity fractions obtained for complementary glycated and reduced glycated peptide mixtures; LC–MS and ESI-HRMS of standard peptides incubated with sodium borohydride in different loading buffer. (DOCX 3.69 MB)

## Data Availability

The data presented in this study are available in the article and the Supplementary Material. The raw data are available on request from the corresponding author.
