# Supplementary material for: Limitations of boronate affinity chromatography for the specific enrichment of fructose-derived early glycation products in protein analytics
Source: Anal Bioanal Chem. 2025 Aug 7;417(23):5239–51. doi: 10.1007/s00216-025-06044-2 (PMC12431902; doi:10.1007/s00216-025-06044-2)
Supplement: Supplementary file 1 — Supplementary Material 1: LC–MS settings; optimized collision energies and source parameters in scheduled MRM; sensitivity and linearity parameters for glycated peptides; XICs of qualifier and quantifier transitions for Heyns peptide spiked plasma samples depleted of Amadori peptides; precursor list and optimized normalized collision energies in direct infusion MS/MS (PRM); recovery rates and percentage distribution of glycated and reduced glycated peptide standards in affinity fractions; raw and annotated tandem mass spectra of unmodified, glycated and reduced glycated peptide standards; RP chromatograms of affinity fractions obtained for complementary glycated and reduced glycated peptide mixtures; LC–MS and ESI-HRMS of standard peptides incubated with sodium borohydride in different loading buffer. (DOCX 3.69 MB) [file 216_2025_6044_MOESM1_ESM.docx]

**Supporting Information**

**Limitations of boronate affinity chromatography for the specific enrichment of fructose-derived early glycation products in protein analytics**

Sebastian Lux, Clara Vogt, Milena Voll, Ralf Hoffmann

**Table of contents**

[Supplementary tables 3](#_Toc197348761)

[Tab. S1 Parameters and settings used for RP-HPLC-ESI-QqLIT-MS. 3](#_Toc197348762)

[Tab. S2 Parameters used for glycated peptides in scheduled MRM. 4](#_Toc197348763)

[Tab. S3 Precursor ions of unmodified, hexose- and hexitol-modified peptides targeted by high resolution ESI-QqOrbitrap-MS (direct infusion) in PRM-mode using individually optimized normalized collision energies (NCE). 5](#_Toc197348764)

[Tab. S4 Instrumental limits of detection (ILD), instrumental limits of quantitation (ILQ), and linearity parameters for a dilution series of seven synthetic Heyns peptides in aqueous buffer using three different MRM transitions per analyte. 6](#_Toc197348765)

[Tab. S5 Recovery rates of glycated and reduced glycated peptide standards in affinity fractions based on their relative peak areas to reference mixtures. 7](#_Toc197348766)

[Tab. S6 Percentage distribution of glycated and reduced glycated peptide standards in affinity fractions. 8](#_Toc197348767)

[Supplementary figures 9](#_Toc197348768)

[Fig. S1 Tandem mass spectra of doubly protonated precursor ions of Amadori (a, *m/z* 1052.5) or Heyns peptide #3 (b, *m/z* 1053.1) acquired on the QTRAP4000 (direct infusion) at a collision energy (CE) of 50%. 9](#_Toc197348769)

[Fig. S2 Peak areas obtained for a dilution series of synthetic Heyns peptide #3 in aqueous buffer using the MRM transitions *m/z* 1053 → 1005 (a, Heyns peptide qualifier) and *m/z* 702 → 1007 (b, quantifier). 10](#_Toc197348770)

[Fig. S3 RP chromatogram of the wash fraction collected in boronate affinity chromatography (BAC) for a peptide mixture containing three fructated (Hey) and three glucated (Ama, 1 nmol each) peptides. 11](#_Toc197348771)

[Fig. S4 RP chromatograms from 20 to 34 min of an SPE-purified peptide mixture (A) containing three fructated (Hey) and three glucated peptides (Ama, 0.75 nmol each) as well as unbound (B-D) and enriched fractions (E-G) collected by BAC. 12](#_Toc197348772)

[Fig. S5 RP chromatograms and ESI-MS (inserts) of SPE-purified peptide mixtures (*n*= 2) containing fructated (Hey) and glucated peptides (Ama, 0.75 nmol each) incubated in the presence (*r*Ama/*r*Hey, b,d) or absence (a,c) of sodium borohydride in ammonium acetate loading buffer (250 mmol/L, 50 mmol/L magnesium acetate, pH 8.1). 13](#_Toc197348773)

[Fig. S6 Zoomed high-resolution mass spectra (*m/z* 900-906) of two peptide mixtures containing different fructated (Hey) and glucated peptides (Ama) incubated in ammonium acetate (c/d, 250 mmol/L, 50 mmol/L magnesium acetate, pH 8.1) or sodium phosphate loading buffer (e/f, 50 mmol/L, pH 8.5) in the absence (a/b) or presence (*r*Ama/*r*Hey, c-f) of sodium borohydride. 14](#_Toc197348774)

[Fig. S7 Tandem mass spectra of doubly protonated precursor ions of the unreduced (a/b, *m/z* 1104.1) and reduced (c, *m/z* 1105.1) Amadori (red) or Heyns peptide #5 (blue) using a normalized collision energy (NCE) of 22. 15](#_Toc197348775)

[Fig. S8 Tandem mass spectra of doubly protonated precursor ions of the reduced Amadori (red, *m/z* 1105.1) and the unmodified peptide #5 (gray, *m/z* 1023.1) at a normalized collision energy (NCE) of 30. 15](#_Toc197348776)

[Fig. S9 Tandem mass spectra recorded for quadruply charged precursor ions detected at *m/z* 625.0297 (a) and *m/z* 666.0468 (c,e) and triply charged precursor ions detected at *m/z* 833.0372 (b) and *m/z* 887.7267 (d,f) of unmodified (a,b), reduced fructated (c,d), and reduced glucated peptide #2 (e,f). 16](#_Toc197348777)

[Fig. S10 Annotated tandem mass spectra recorded for quadruply charged precursor ions detected at *m/z* 625.0297 (a) and *m/z* 666.0468 (c,e) and triply charged precursor ions detected at *m/z* 833.0372 (b) and *m/z* 887.7267 (d,f) of unmodified (a,b), reduced fructated (c,d), and reduced glucated peptide #2 (e,f). 17](#_Toc197348778)

[Fig. S11 Tandem mass spectra recorded for triply charged precursor ions detected at *m/z* 647.9789 (a) and *m/z* 702.6684 (c,e) and doubly charged precursor ions detected at *m/z* 971.4648 (b) and *m/z* 1053.4990 (d,f) of unmodified (a,b), reduced fructated (c,d), and reduced glucated peptide #3 (e,f). 18](#_Toc197348779)

[Fig. S12 Annotated tandem mass spectra recorded for triply charged precursor ions detected at *m/z* 647.9789 (a) and *m/z* 702.6684 (c,e) and doubly charged precursor ions detected at *m/z* 971.4648 (b) and *m/z* 1053.4990 (d,f) of unmodified (a,b), reduced fructated (c,d), and reduced glucated peptide #3 (e,f). 19](#_Toc197348780)

[Fig. S13 Tandem mass spectra recorded for triply charged precursor ions detected at *m/z* 547.3174 (a) and *m/z* 602.0069 (c,e) and doubly charged precursor ions detected at *m/z* 820.4725 (b) and *m/z* 902.5067 (d,f) of unmodified (a,b), reduced fructated (c,d), and reduced glucated peptide #4 (e,f). 20](#_Toc197348781)

[Fig. S14 Annotated tandem mass spectra recorded for triply charged precursor ions detected at *m/z* 547.3174 (a) and *m/z* 602.0069 (c,e) and doubly charged precursor ions detected at *m/z* 820.4725 (b) and *m/z* 902.5067 (d,f) of unmodified (a,b), reduced fructated (c,d), and reduced glucated peptide #4 (e,f). 21](#_Toc197348782)

[Fig. S15 Tandem mass spectra recorded for triply charged precursor ions detected at *m/z* 682.3700 (a) and *m/z* 737.0595 (c,e) and doubly charged precursor ions detected at *m/z* 1023.0513 (b) and *m/z* 1105.0856 (d,f) of unmodified (a,b), reduced fructated (c,d), and reduced glucated peptide #5 (e,f). 22](#_Toc197348783)

[Fig. S16 Annotated tandem mass spectra recorded for triply charged precursor ions detected at *m/z* 682.3700 (a) and *m/z* 737.0595 (c,e) and doubly charged precursor ions detected at *m/z* 1023.0513 (b) and *m/z* 1105.0856 (d,f) of unmodified (a,b), reduced fructated (c,d), and reduced glucated peptide #5 (e,f). 23](#_Toc197348784)

[Fig. S17 Tandem mass spectra recorded for triply charged precursor ions detected at *m/z* 550.9698 (a) and *m/z* 605.6593 (c,e) and doubly charged precursor ions detected at *m/z* 825.9511 (b) and *m/z* 907.9853 (d,f) of unmodified (a,b), reduced fructated (c,d), and reduced glucated peptide #6 (e,f). 24](#_Toc197348785)

[Fig. S18 Annotated tandem mass spectra recorded for triply charged precursor ions detected at *m/z* 550.9698 (a) and *m/z* 605.6593 (c,e) and doubly charged precursor ions detected at *m/z* 825.9511 (b) and *m/z* 907.9853 (d,f) of unmodified (a,b), reduced fructated (c,d), and reduced glucated peptide #6 (e,f). 25](#_Toc197348786)

[Fig. S19 Tandem mass spectra recorded for triply charged precursor ions detected at *m/z* 614.3098 (a) and *m/z* 668.9993 (c,e) and doubly charged precursor ions detected at *m/z* 920.9611 (b) and *m/z* 1002.9954 (d,f) of unmodified (a,b), reduced fructated (c,d), and reduced glucated peptide #7 (e,f). 26](#_Toc197348787)

[Fig. S20 Annotated tandem mass spectra recorded for triply charged precursor ions detected at *m/z* 614.3098 (a) and *m/z* 668.9993 (c,e) and doubly charged precursor ions detected at *m/z* 920.9611 (b) and *m/z* 1002.9954 (d,f) of unmodified (a,b), reduced fructated (c,d), and reduced glucated peptide #7 (e,f). 27](#_Toc197348788)

[Fig. S21 RP chromatograms from 18 to 36 min of an SPE-purified peptide mixture containing three fructated (Hey) and three glucated peptides (Ama, 1 nmol each) incubated in sodium phosphate buffer (50 mmol/L, pH 8.5) in the presence (orange, *r*Ama/*r*Hey, a) or absence (blue, b) of sodium borohydride and the corresponding elution (c,d) and wash fractions (e,f) obtained by BAC using ammonium acetate loading buffer (250 mmol/L, 50 mmol/L magnesium acetate, pH 8.1). 28](#_Toc197348789)

[Fig. S22 RP chromatograms from 20 to 34 min of an SPE-purified peptide mixture (A) containing three fructated (Heyns) and three glucated peptides (Amadori, *n*=3, 1 nmol each) reduced with sodium borohydride and the wash (B-D) and enriched fractions (E-G) collected by BAC. 29](#_Toc197348790)

# Supplementary tables

## Tab. S1 Parameters and settings used for RP-HPLC-ESI-QqLIT-MS.

| **Parameter** | **Settings** |
| --- | --- |
| **ESI-source parameters** | |
| **IS [V]** | 5500 |
| **TEM [°C]** | 650 |
| **Nebulizer gas (Gas 1, psig)** | 40 |
| **Drying gas (Gas 2, psig)** | 75 |
| **Curtain gas (CUR, psig)** | 35 |
| **Mass analyzer settings** | |
| **Declustering potential (DP, V)** | ^a^ |
| **Collision cell entrance potential (EP, V)** | 10 |
| **Collision cell exit potential (CXP, V)** | ^a^ |
| **Interface heating** | on |
| **Q_1_ resolution** | unit |
| **Q_3_ resolution** | - |
| **Collision (CAD) gas** | high |
| **Collision potential (CE, V)** | ^a^ |
| **Cycle time [s]** | 1.35 |
| **Dwell time [ms]** | ^b^ |

^a^: Analyte and charge state dependent parameters (see Tab. S2), ^b^: Automatically defined by the Scheduled MRM™ Algorithm.

## Tab. S2 Parameters used for glycated peptides in scheduled MRM.

| **#** | **Sequence** | **RT**  **[min]** | ***m/z* [M+*n*H]*^n^*^+^** | ***n*** | **Q_1_/Q_3_ masses** | **Fragment ion** | **DP**  **[V]** | **EP**  **[V]** | **CE [V]** | **CXP [V]** |
| --- | --- | --- | --- | --- | --- | --- | --- | --- | --- | --- |
| 1 | AVGDK_Hex_LPECEAVC*GKPK | 9.2 | 674.0 | 3 | 674 → 656 | [M+3H-3H_2_O]^3+^ | 88 | 10 | 29 | 8 |
|  |  |  |  |  | **674 → 642** | [M+3H-2H_2_O-C_2_H_4_O_2_]^3+^ |  |  | 30 | 10 |
|  |  |  |  |  | **674 → 759** | y_7_^+^ |  |  | 37 | 12 |
| 2 | TC*VADESAENC*DK_Hex_SLHTLFGDK | 13.3 | 665.5 | 4 | **666 → 642** | [M+4H-2H_2_O-C_2_H_4_O_2_]^4+^ | 107 | 10 | 29 | 9 |
|  |  |  | 887.1 | 3 | 887 → 881 | [M+3H-H_2_O]^3+^ |  |  | 33 | 12 |
|  |  |  |  |  | **887 → 1018** | y_9_^+^ |  |  | 53 | 16 |
| 3 | ADLAK_Hex_YIC*ENQDSISSK | 13.5 | 1052.5 | 2 | 1053 → 1026 | [M+2H-3H_2_O]^2+^ | 113 | 10 | 49 | 16 |
|  |  |  |  |  | **1053 → 1005** | [M+2H-2H_2_O-C_2_H_4_O_2_]^2+^ |  |  | 50 | 15 |
|  |  |  | 702.0 | 3 | **702 → 1007** | y_9_^+^ |  |  | 35 | 16 |
| 4 | K_Hex_VPQVSTPTLVEVSR | 14.6 | 901.5 | 2 | **902 → 854** | [M+2H-2H_2_O-C_2_H_4_O_2_]^2+^ | 90 | 10 | 44 | 12 |
|  |  |  | 601.3 | 3 | 601 → 70 | Pro_Im_ |  |  | 109 | 2 |
|  |  |  |  |  | **601 → 901** | y_8_^+^ |  |  | 29 | 14 |
| 5 | VFDEFK_Hex_PLVEEPQNLIK | 18.5 | 1104.1 | 2 | 1104 → 1077 | [M+2H-3H_2_O]^2+^ | 141 | 10 | 51 | 16 |
|  |  |  |  |  | **1104 → 1056** | [M+2H-2H_2_O-C_2_H_4_O_2_]^2+^ |  |  | 57 | 17 |
|  |  |  | 736.4 | 3 | **736 → 981** | y_15_^2+^ |  |  | 29 | 14 |
| 6 | AEFAEVSK_Hex_LVTDLTK | 18.6 | 907.0 | 2 | 907 → 880 | [M+2H-3H_2_O]^2+^ | 108 | 10 | 43 | 14 |
|  |  |  |  |  | **907 → 859** | [M+2H-2H_2_O-C_2_H_4_O_2_]^2+^ |  |  | 44 | 13 |
|  |  |  | 605.0 | 3 | **605 → 676** | y_6_^+^ |  |  | 21 | 10 |
| 7 | EQLK_Hex_AVMDDFAAFVEK | 18.7 | 1002.0 | 2 | **1002 → 954** | [M+2H-2H_2_O-C_2_H_4_O_2_]^2+^ | 89 | 10 | 47 | 15 |
|  |  |  | 668.3 | 3 | 668 → 120 | Phe_Im_ |  |  | 89 | 6 |
|  |  |  |  |  | **668 → 1042** | y_9_^+^ |  |  | 29 | 16 |

C^*^: carbamidomethylated cysteine, K_Hex_: hexosamine-modified lysine, red – quantifier (peptide backbone fragment), blue – qualifier (96 Da neutral loss)

## Tab. S3 Precursor ions of unmodified, hexose- and hexitol-modified peptides targeted by high resolution ESI-QqOrbitrap-MS (direct infusion) in PRM-mode using individually optimized normalized collision energies (NCE).

| **#** | **Protein/Location** | **Sequence** | ***m/z*  [M+*n*H]*^n^*^+^** | ***n*** | **NCE [%]** |
| --- | --- | --- | --- | --- | --- |
| 2 | HSA  T_52_ – **K**_64_ – K_73_ | TC*****VADESAENC*****D**K**SLHTLFGDK | 833.0372 | 3 | 24 |
|  |  |  | 625.0297 | 4 | 22 |
|  |  | TC*****VADESAENC*****D**K_Hexose_**SLHTLFGDK | 887.0548 | 3 | 18 |
|  |  |  | 665.5429 | 4 | 18 |
|  |  | TC*****VADESAENC*****D**K_Hexitol_**SLHTLFGDK | 887.7267 | 3 | 24 |
|  |  |  | 666.0468 | 4 | 22 |
| 3 | HSA  A_258_ – **K**_262_ – K_274_ | ADLA**K**YIC*****ENQDSISSK | 971.4648 | 2 | 30 |
|  |  |  | 647.9789 | 3 | 20 |
|  |  | ADLA**K_Hexose_**YIC*****ENQDSISSK | 1052.4912 | 2 | 22 |
|  |  |  | 701.9965 | 3 | 26 |
|  |  | ADLA**K_Hexitol_**YIC*****ENQDSISSK | 1053.4990 | 2 | 32 |
|  |  |  | 702.6684 | 3 | 20 |
| 4 | HSA  **K**_414_ – R_428_ | **K**VPQVSTPTLVEVSR | 820.4725 | 2 | 30 |
|  |  |  | 547.3174 | 3 | 20 |
|  |  | **K_Hexose_**VPQVSTPTLVEVSR | 901.4989 | 2 | 20 |
|  |  |  | 601.3350 | 3 | 20 |
|  |  | **K_Hexitol_**VPQVSTPTLVEVSR | 902.5067 | 2 | 34 |
|  |  |  | 602.0069 | 3 | 20 |
| 5 | HSA  V_373_ – **K**_378_ – K_389_ | VFDEF**K**PLVEEPQNLIK | 1023.0513 | 2 | 30 |
|  |  |  | 682.3700 | 3 | 20 |
|  |  | VFDEF**K_Hexose_**PLVEEPQNLIK | 1104.0777 | 2 | 22 |
|  |  |  | 736.3876 | 3 | 20 |
|  |  | VFDEF**K_Hexitol_**PLVEEPQNLIK | 1105.0856 | 2 | 32 |
|  |  |  | 737.0595 | 3 | 20 |
| 6 | HSA  A_226_ – **K**_233_ – K_240_ | AEFAEVS**K**LVTDLTK | 825.9511 | 2 | 30 |
|  |  |  | 550.9698 | 3 | 20 |
|  |  | AEFAEVS**K_Hexose_**LVTDLTK | 906.9775 | 2 | 22 |
|  |  |  | 604.9874 | 3 | 20 |
|  |  | AEFAEVS**K_Hexitol_**LVTDLTK | 907.9853 | 2 | 32 |
|  |  |  | 605.6593 | 3 | 20 |
| 7 | HSA  E_542_ – **K**_545_ – K_557_ | EQL**K**AVMDDFAAFVEK | 920.9611 | 2 | 30 |
|  |  |  | 614.3098 | 3 | 20 |
|  |  | EQL**K_Hexose_**AVMDDFAAFVEK | 1001.9875 | 2 | 20 |
|  |  |  | 668.3274 | 3 | 14 |
|  |  | EQL**K_Hexitol_**AVMDDFAAFVEK | 1002.9954 | 2 | 32 |
|  |  |  | 668.9993 | 3 | 20 |

Hexitol-lysine: +164.0685 Da (C_6_H_12_O_5_); Hexosyl-lysine: +162.0528 Da (C_6_H_10_O_5_); C^*^: carbamidomethylated cysteine

## Tab. S4 Instrumental limits of detection (ILD), instrumental limits of quantitation (ILQ), and linearity parameters for a dilution series of seven synthetic Heyns peptides in aqueous buffer using three different MRM transitions per analyte.

| **#** | **Sequence** | ***m/z* [M+*n*H]*^n^*^+^** | ***n*** | **Q_1_/Q_3_ masses** | **ILD**  **[pmol]** | **ILQ**  **[pmol]** | **Peak Area ILQ** | **Range [pmol]** | **LDR** | **R^2^** |
| --- | --- | --- | --- | --- | --- | --- | --- | --- | --- | --- |
| 1 | AVGDK_Hey_LPECEAVC*GKPK | 674 | 3 | 674 → 656 | 0.5 | 1 | 9.25E+03 | 2.5 – 100 | 40 | 0.998 |
|  |  |  |  | **674 → 642** | 0.5 | 1 | 8.94E+03 | 2.5 – 100 | 40 | 0.998 |
|  |  |  |  | **674 → 759** | 1 | 2.5 | 1.66E+04 | 2.5 – 100 | 40 | 0.998 |
| 2 | TC*VADESAENC*DK_Hey_SLHTLFGDK | 665.5 | 4 | **666 → 642** | 0.25 | 1 | 9.55E+03 | 2.5 – 100 | 40 | 0.992 |
|  |  | 887 | 3 | 887 → 881 | 0.25 | 0.5 | 3.88E+03 | 5 – 50 | 10 | 0.999 |
|  |  |  |  | **887 → 1018** | 1 | 5 | 4.96E+03 | 10 – 100 | 10 | 0.994 |
| 3 | ADLAK_Hey_YIC*ENQDSISSK | 1052.5 | 2 | 1053 → 1026 | 2.5 | 5 | 1.86E+04 | 5 – 100 | 20 | 0.992 |
|  |  |  |  | **1053 → 1005** | 1 | 2.5 | 6.02E+03 | 2.5 – 100 | 40 | 0.991 |
|  |  | 702 | 3 | **702 → 1007** | 0.5 | 1 | 5.52E+03 | 1 – 100 | 100 | 1.000 |
| 4 | K_Hey_VPQVSTPTLVEVSR | 901.5 | 2 | **902 → 854** | 1 | 2.5 | 1.21E+04 | 2.5 – 100 | 40 | 0.995 |
|  |  | 601.3 | 3 | 601 → 70 | 0.25 | 0.5 | 3.96E+04 | 1 – 50 | 50 | 0.993 |
|  |  |  |  | **601 → 901** | 0.25 | 0.5 | 2.57E+04 | 1 – 50 | 50 | 0.995 |
| 5 | VFDEFK_Hey_PLVEEPQNLIK | 1104.1 | 2 | 1104 → 1077 | 2.5 | 5 | 2.13E+03 | 10 – 100 | 10 | 0.977 |
|  |  |  |  | **1104 → 1056** | 5 | 10 | 1.01E+04 | 10 – 100 | 10 | 0.979 |
|  |  | 736.4 | 3 | **736 → 981** | 2.5 | 5 | 5.78E+03 | 5 – 100 | 20 | 0.986 |
| 6 | AEFAEVSK_Hey_LVTDLTK | 907 | 2 | 907 → 880 | 2.5 | 5 | 1.12E+04 | 5 – 100 | 20 | 0.981 |
|  |  |  |  | **907 → 859** | 1 | 2.5 | 2.31E+03 | 5 – 100 | 20 | 0.982 |
|  |  | 605 | 3 | **605 → 676** | 2.5 | 5 | 2.57E+04 | 5 – 100 | 20 | 0.991 |
| 7 | EQLK_Hey_AVMDDFAAFVEK | 1002 | 2 | **1002 → 954** | 5 | 10 | 9.27E+03 | 10 – 100 | 10 | 0.947 |
|  |  | 668.3 | 3 | 668 → 120 | 1 | 2.5 | 1.54E+04 | 2.5 – 100 | 40 | 0.975 |
|  |  |  |  | **668 → 1042** | 2.5 | 5 | 2.05E+04 | 5 – 100 | 20 | 0.974 |

ILDs and ILQs were defined by signal-to-noise ratios (S/N) better than 3 and ≥ 10, respectively. C^*^: carbamidomethylated cysteine, K_Hey_: gluco/mannosamine-modified lysine, LDR: linear dynamic range, R^2^: coefficient of determination, red – quantifier (peptide backbone fragment), blue – qualifier (96 Da neutral loss)

## Tab. S5 Recovery rates of glycated and reduced glycated peptide standards in affinity fractions based on their relative peak areas to reference mixtures.

The composition of complementary peptide mixtures in each experiment is summarized by the colors orange and purple, denoting mix 1 and mix 2.

| **#** | **Mod** | **Exp 1** 12.5 pmol  NH_4_Ac  pH 8.1 | **Exp 2** 125 pmol  NH_4_Ac  pH 8.1 | **Exp 3** 6 nmol  NH_4_Ac  pH 8.1 | | | **Exp 4** 3 nmol  NH_4_Ac  pH 8.1 | | **Exp 5** 3 nmol  NH_4_Ac  pH 10 | | **Exp 6** 3 nmol  Na-PB  pH 8.5 | | **Exp 7** 3 nmol  NH_4_Ac  pH 8.1 | | **Exp 8** 3 nmol  NH_4_Ac  pH 8.1 | | **Exp 9** 3 nmol  NH_4_Ac  pH 10 | | **Exp 10** 3 nmol  Na-PB  pH 8.5 | |
| --- | --- | --- | --- | --- | --- | --- | --- | --- | --- | --- | --- | --- | --- | --- | --- | --- | --- | --- | --- | --- |
|  |  | Elu  [%] | Elu  [%] | FT [%] | Wash  [%]*^3^ | Elu  [%] | FT/W  [%] | Elu  [%] | FT/W [%] | Elu  [%] | FT/W [%] | Elu  [%] | FT/W [%] | Elu  [%] | FT/W [%] | Elu  [%] | FT/W [%] | Elu  [%] | FT/W [%] | Elu  [%] |
| 1 | Ama | 75*^1^ |  | - | - | 75 |  |  |  |  |  |  |  |  |  |  |  |  |  |  |
|  | *r*Ama |  |  |  |  |  |  |  |  |  |  |  |  |  |  |  |  |  |  |  |
|  | Hey | < LOQ | 0.3 | 5.6 | 87 | - |  |  |  |  |  |  |  |  |  |  |  |  |  |  |
|  | *r*Hey |  |  |  |  |  |  |  |  |  |  |  |  |  |  |  |  |  |  |  |
| 2 | Ama | 72*^1^ |  |  |  |  | - | 146 | - | 143 | <5*^2^ | 146 | - | 105 |  |  |  |  |  |  |
|  | *r*Ama |  |  |  |  |  |  |  |  |  |  |  | - | 93 | 6 | 134 | 3 | 137 | 112 | 24 |
|  | Hey | < LOQ | 1.6 |  |  |  | 132 | 8.7 | 144 | 9.0 | 133 | 6.3 | 106 | 9.2 |  |  |  |  |  |  |
|  | *r*Hey |  |  |  |  |  |  |  |  |  |  |  | - | 86 | 15 | 138 | - | 135 | 155 | 9 |
| 3 | Ama | 84*^1^ |  | - | - | 89 | - | 152 | - | 120 | <3*^2^ | 154 | 5.1 | 95 |  |  |  |  |  |  |
|  | *r*Ama |  |  |  |  |  |  |  |  |  |  |  | 9.1 | 93 | 8 | 143 | 4 | 131 | 67 | 48 |
|  | Hey | 5.3 | 4.0 | 4.6 | 69 | 5.4 | 110 | 6.5 | 112 | 5.7 | 142 | 3.6 | 83 | 1.8 |  |  |  |  |  |  |
|  | *r*Hey |  |  |  |  |  |  |  |  |  |  |  | - | 77 | 21 | 134 | 4 | 123 | 110 | 38 |
| 4 | Ama | 93*^1^ |  | - | - | 78 | - | 152 | - | 159 | - | 131 | 1.2 | 101 |  |  |  |  |  |  |
|  | *r*Ama |  |  |  |  |  |  |  |  |  |  |  | - | 95 | 9 | 148 | 4 | 145 | 24 | 127 |
|  | Hey | 6.2 | 6.1 | 3.4 | 75 | 6.4 | 152 | 8.7 | 150 | 8.1 | 109 | 7.1 | 76 | 3.1 |  |  |  |  |  |  |
|  | *r*Hey |  |  |  |  |  |  |  |  |  |  |  | - | 90 | 20 | 140 | 3 | 137 | 21 | 137 |
| 5 | Ama | 81*^1^ |  | - | - | 58 | - | 226 | - | 187 | 51 | 134 | - | 111 |  |  |  |  |  |  |
|  | *r*Ama |  |  |  |  |  |  |  |  |  |  |  | 10 | 51 | 52 | 135 | 16 | 116 | 237 | - |
|  | Hey | < LOQ | 1.6 | 2.3 | 51 | 1.3 | 140 | - | 134 | 1.1 | 205 | 1.0 | 92 | - |  |  |  |  |  |  |
|  | *r*Hey |  |  |  |  |  |  |  |  |  |  |  | 19 | 58 | 51 | 110 | 19 | 135 | 171 | 3 |
| 6 | Ama | 98*^1^ |  | - | - | 64 | - | 193 | - | 145 | 9 | 144 | - | 98 |  |  |  |  |  |  |
|  | *r*Ama |  |  |  |  |  |  |  |  |  |  |  | 11 | 57 | 57 | 166 | 21 | 146 | 211 | 73 |
|  | Hey | 7.8 | 1.6 | 2.1 | 23 | <9*^2^ | 131 | - | 138 | 2.8 | 197 | 2.7 | 101 | - |  |  |  |  |  |  |
|  | *r*Hey |  |  |  |  |  |  |  |  |  |  |  | 19 | 59 | 62 | 140 | 24 | 166 | 191 | 11 |
| 7 | Ama | 107*^1^ |  | - | - | 71 | - | 251 | - | 195 | 19 | 176 | - | 118 |  |  |  |  |  |  |
|  | *r*Ama |  |  |  |  |  |  |  |  |  |  |  | 3.8 | 41 | -*^2^ | -*^2^ | -*^2^ | -*^2^ | -*^2^ | -*^2^ |
|  | Hey | 7.3 | 1.7 | 3.1 | 26 | 1.7 | 170 | - | 185 | - | 418 | 3.1 | 81 | - |  |  |  |  |  |  |
|  | *r*Hey |  |  |  |  |  |  |  |  |  |  |  | 8.4 | 56 | -*^2^ | -*^2^ | -*^2^ | -*^2^ | -*^2^ | -*^2^ |

*^1^: normalized recovery after UV/VIS adjustment to Heyns peptides; *^2^: overlap with other signals in UV; *^3^: after SPE

## Tab. S6 Percentage distribution of glycated and reduced glycated peptide standards in affinity fractions.

The composition of complementary peptide mixtures in each experiment is summarized by the colors orange and purple, denoting mix 1 and mix 2.

| **#** | **Mod** | **Exp 3** 6 nmol  NH_4_Ac  pH 8.1 | | | **Exp 4** 3 nmol  NH_4_Ac  pH 8.1 | | **Exp 5** 3 nmol  NH_4_Ac  pH 10 | | **Exp 6** 3 nmol  Na-PB  pH 8.5 | | **Exp 7** 3 nmol  NH_4_Ac  pH 8.1 | | **Exp 8** 3 nmol  NH_4_Ac  pH 8.1 | | **Exp 9** 3 nmol  NH_4_Ac  pH 10 | | **Exp 10** 3 nmol  Na-PB  pH 8.5 | |
| --- | --- | --- | --- | --- | --- | --- | --- | --- | --- | --- | --- | --- | --- | --- | --- | --- | --- | --- |
|  |  | FT [%] | Wash  [%]*^3^ | Elu  [%] | FT/W  [%] | Elu  [%] | FT/W [%] | Elu  [%] | FT/W [%] | Elu  [%] | FT/W [%] | Elu  [%] | FT/W [%] | Elu  [%] | FT/W [%] | Elu  [%] | FT/W [%] | Elu  [%] |
| 1 | Ama | 0 | 0 | 100 |  |  |  |  |  |  |  |  |  |  |  |  |  |  |
|  | *r*Ama |  |  |  |  |  |  |  |  |  |  |  |  |  |  |  |  |  |
|  | Hey | 6 | 94 | 0 |  |  |  |  |  |  |  |  |  |  |  |  |  |  |
|  | *r*Hey |  |  |  |  |  |  |  |  |  |  |  |  |  |  |  |  |  |
| 2 | Ama |  |  |  | 0 | 100 | 0 | 100 | <3 | >97 | 0 | 100 |  |  |  |  |  |  |
|  | *r*Ama |  |  |  |  |  |  |  |  |  | 0 | 100 | 4 | 96 | 2 | 98 | 82 | 18 |
|  | Hey |  |  |  | 94 | 6 | 94 | 6 | 95 | 5 | 92 | 8 |  |  |  |  |  |  |
|  | *r*Hey |  |  |  |  |  |  |  |  |  | 0 | 100 | 10 | 90 | 0 | 100 | 95 | 5 |
| 3 | Ama | 0 | 0 | 100 | 0 | 100 | 0 | 100 | <2 | >98 | 5 | 95 |  |  |  |  |  |  |
|  | *r*Ama |  |  |  |  |  |  |  |  |  | 9 | 91 | 5 | 95 | 3 | 97 | 58 | 42 |
|  | Hey | 6 | 87 | 7 | 94 | 6 | 95 | 5 | 98 | 2 | 98 | 2 |  |  |  |  |  |  |
|  | *r*Hey |  |  |  |  |  |  |  |  |  | 0 | 100 | 14 | 86 | 3 | 97 | 74 | 26 |
| 4 | Ama | 0 | 0 | 100 | 0 | 100 | 0 | 100 | 0 | 100 | 1 | 99 |  |  |  |  |  |  |
|  | *r*Ama |  |  |  |  |  |  |  |  |  | 0 | 100 | 6 | 94 | 3 | 97 | 16 | 84 |
|  | Hey | 4 | 88 | 8 | 95 | 5 | 95 | 5 | 94 | 6 | 96 | 4 |  |  |  |  |  |  |
|  | *r*Hey |  |  |  |  |  |  |  |  |  | 0 | 100 | 13 | 88 | 2 | 98 | 13 | 87 |
| 5 | Ama | 0 | 0 | 100 | 0 | 100 | 0 | 100 | 28 | 72 | 0 | 100 |  |  |  |  |  |  |
|  | *r*Ama |  |  |  |  |  |  |  |  |  | 16 | 84 | 28 | 72 | 12 | 88 | 100 | 0 |
|  | Hey | 4 | 93 | 2 | 100 | 0 | 99 | 1 | 100 | 0 | 100 | 0 |  |  |  |  |  |  |
|  | *r*Hey |  |  |  |  |  |  |  |  |  | 25 | 75 | 32 | 68 | 12 | 88 | 98 | 2 |
| 6 | Ama | 0 | 0 | 100 | 0 | 100 | 0 | 100 | 6 | 94 | 0 | 100 |  |  |  |  |  |  |
|  | *r*Ama |  |  |  |  |  |  |  |  |  | 16 | 84 | 26 | 74 | 13 | 87 | 74 | 26 |
|  | Hey | >6 | >67 | <26 | 100 | 0 | 98 | 2 | 99 | 1 | 100 | 0 |  |  |  |  |  |  |
|  | *r*Hey |  |  |  |  |  |  |  |  |  | 24 | 76 | 31 | 69 | 13 | 87 | 95 | 5 |
| 7 | Ama | 0 | 0 | 100 | 0 | 100 | 0 | 100 | 10 | 90 | 0 | 100 |  |  |  |  |  |  |
|  | *r*Ama |  |  |  |  |  |  |  |  |  | 8 | 92 |  |  |  |  |  |  |
|  | Hey | 10 | 84 | 6 | 100 | 0 | 100 | 0 | 99 | 1 | 100 | 0 |  |  |  |  |  |  |
|  | *r*Hey |  |  |  |  |  |  |  |  |  | 13 | 87 |  |  |  |  |  |  |

*^2^: overlap with other signals in UV; *^3^: after SPE

# Supplementary figures


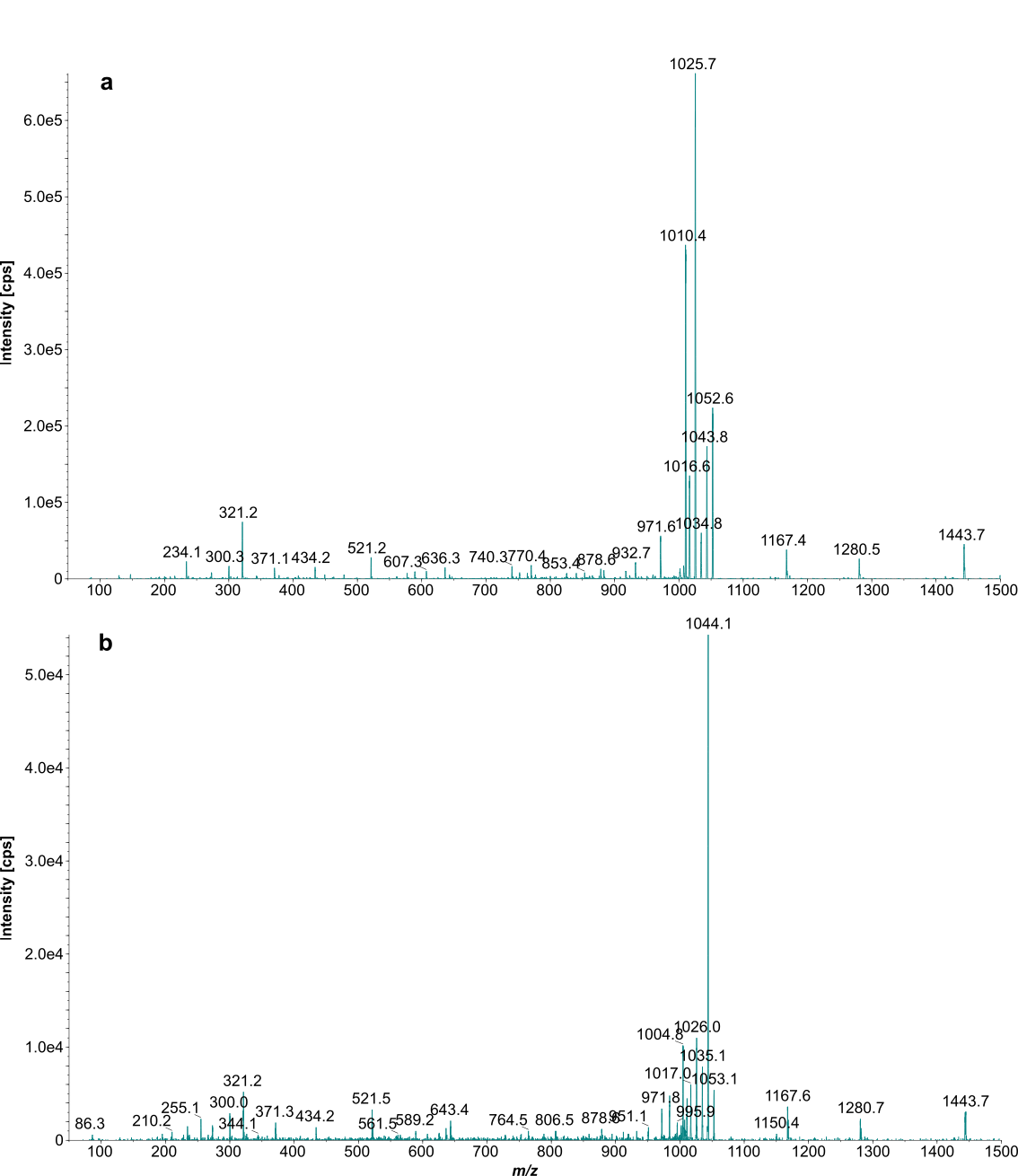


## **Fig. S1** Tandem mass spectra of doubly protonated precursor ions of Amadori (a, *m/z* 1052.5) or Heyns peptide #3 (b, *m/z* 1053.1) acquired on the QTRAP4000 (direct infusion) at a collision energy (CE) of 50%.


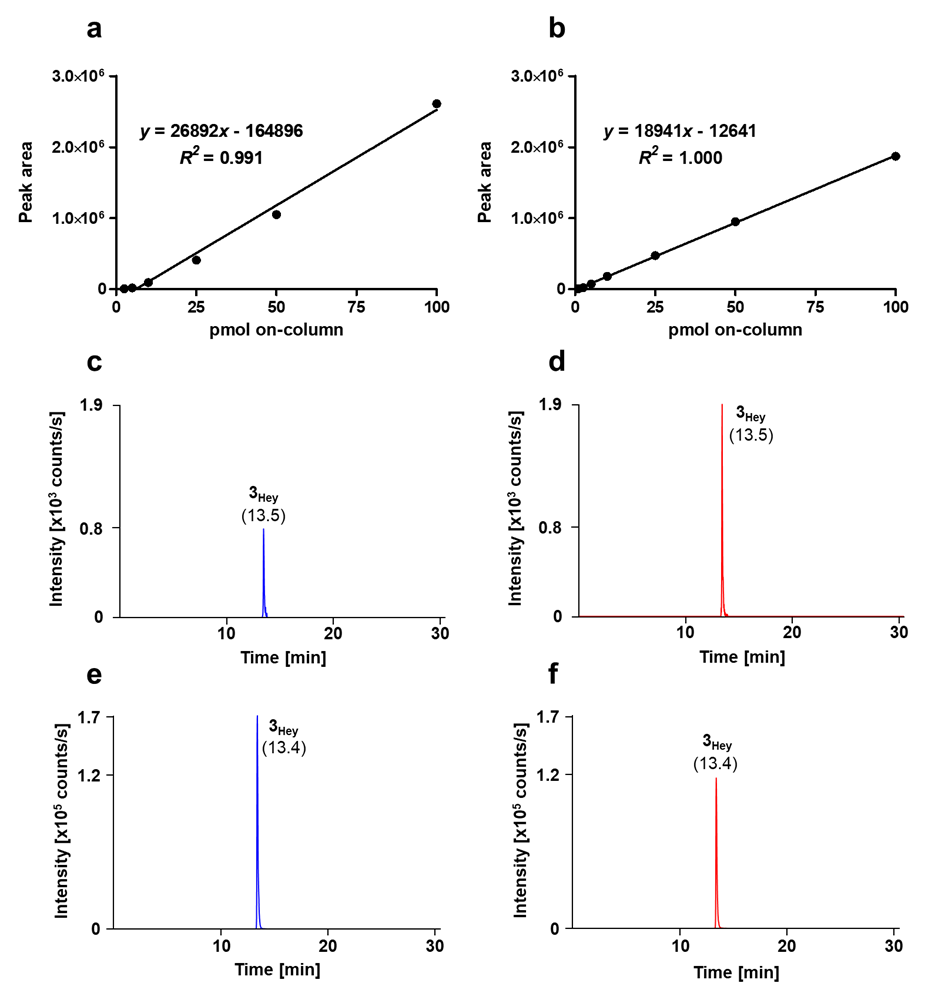


## **Fig. S2** Peak areas obtained for a dilution series of synthetic Heyns peptide #3 in aqueous buffer using the MRM transitions *m/z* 1053 → 1005 (a, Heyns peptide qualifier) and *m/z* 702 → 1007 (b, quantifier).

XICs of the qualifier (blue, c/e) and quantifier (red, d/f) are shown for a spiked plasma sample depleted of Amadori peptides by BAC with on-column loads of 1 pmol (c/d) and 100 pmol (e/f).


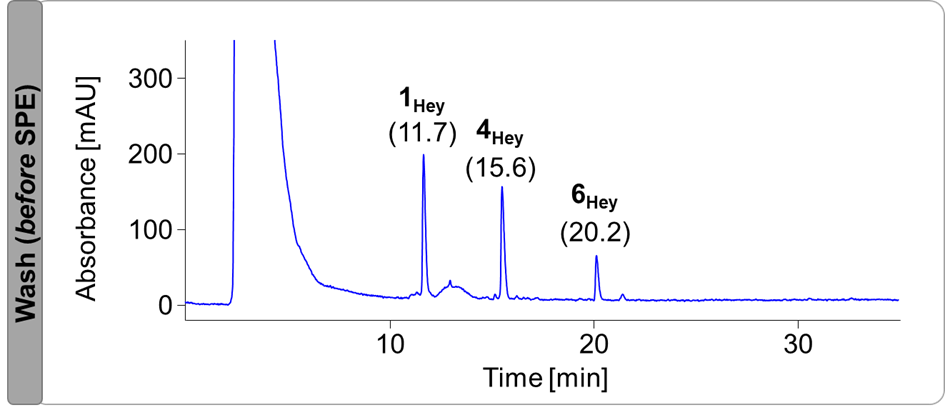


## **Fig. S3** RP chromatogram of the wash fraction collected in boronate affinity chromatography (BAC) for a peptide mixture containing three fructated (Hey) and three glucated (Ama, 1 nmol each) peptides.

The peptides were separated on a Jupiter C_18_ column at 60°C using a linear gradient from 95% eluent A to 95% eluent B in 30 min. The absorbance was recorded at 214 nm. Peptide sequences and modification sites are listed in Tab. S3. Recovery rates and percentage distribution in affinity processed fractions are summarized in Tab. S5 (Exp 3).


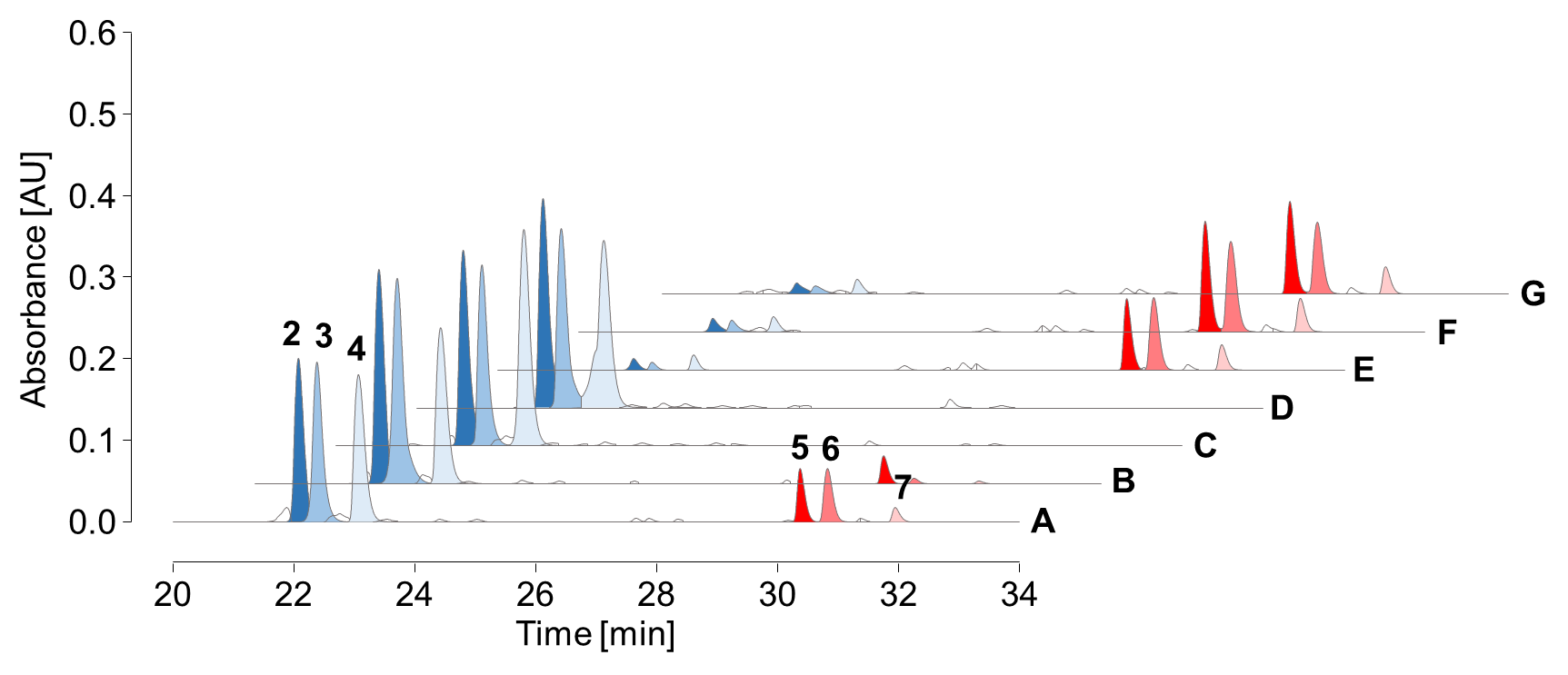


## **Fig. S4** RP chromatograms from 20 to 34 min of an SPE-purified peptide mixture (A) containing three fructated (Hey) and three glucated peptides (Ama, 0.75 nmol each) as well as unbound (B-D) and enriched fractions (E-G) collected by BAC.

Equilibration, sample loading, and column washing in affinity chromatography were performed using either sodium phosphate (50 mmol/L, pH 8.5, B/E) or ammonium acetate loading buffer (250 mmol/L, 50 mmol/L magnesium acetate, pH 8.1, C/F), with additional equilibration at basic pH (pH 10, D/G) tested for the latter. Peptides were separated on an Aqua C_18_ column at 60°C using a linear gradient from 97% eluent A to 40% eluent C in 37 min. The absorbance was recorded at 214 nm. Peptide sequences and modification sites are listed in Tab. S3. Recovery rates and percentage distribution in affinity processed fractions are summarized in Tab. S5 and Tab. S6 (Exp 4/Exp 5/Exp 6).


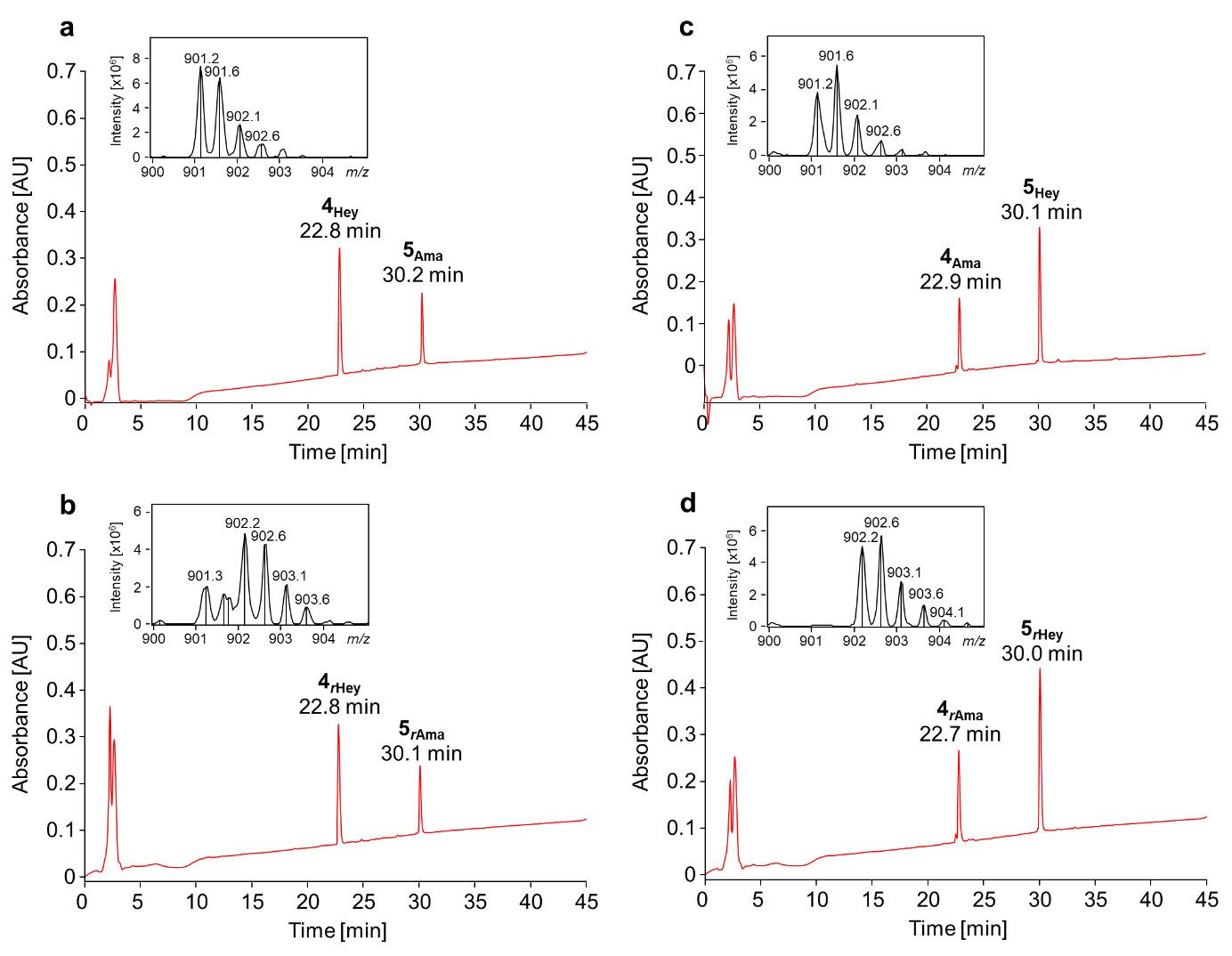


## Fig. S5 RP chromatograms and ESI-MS (inserts) of SPE-purified peptide mixtures (*n*= 2) containing fructated (Hey) and glucated peptides (Ama, 0.75 nmol each) incubated in the presence (*r*Ama/*r*Hey, b,d) or absence (a,c) of sodium borohydride in ammonium acetate loading buffer (250 mmol/L, 50 mmol/L magnesium acetate, pH 8.1).

Peptides were separated on an Aqua C_18_ column at 60°C using a linear gradient from 97% eluent A to 40% eluent C in 37 min. Absorbance was recorded at 214 nm. Mass spectra were recorded online from 22.6 min to 24.1 min (a), 22.7 min to 24.0 min (b,d) and 22.7 min to 24.1 min (c) on an ESI-ion trap-MS in positive ion mode from *m/z* 500 to 1200. The small insets show the isotopic patterns of the doubly protonated precursor ions of glycated peptide #4 (K_Hex_VPQVSTPTLVEVSR, *m/z* 901.5) and its reduced version (*m/z* 902.5). Peptide sequences and modification sites are listed in Tab. S3.


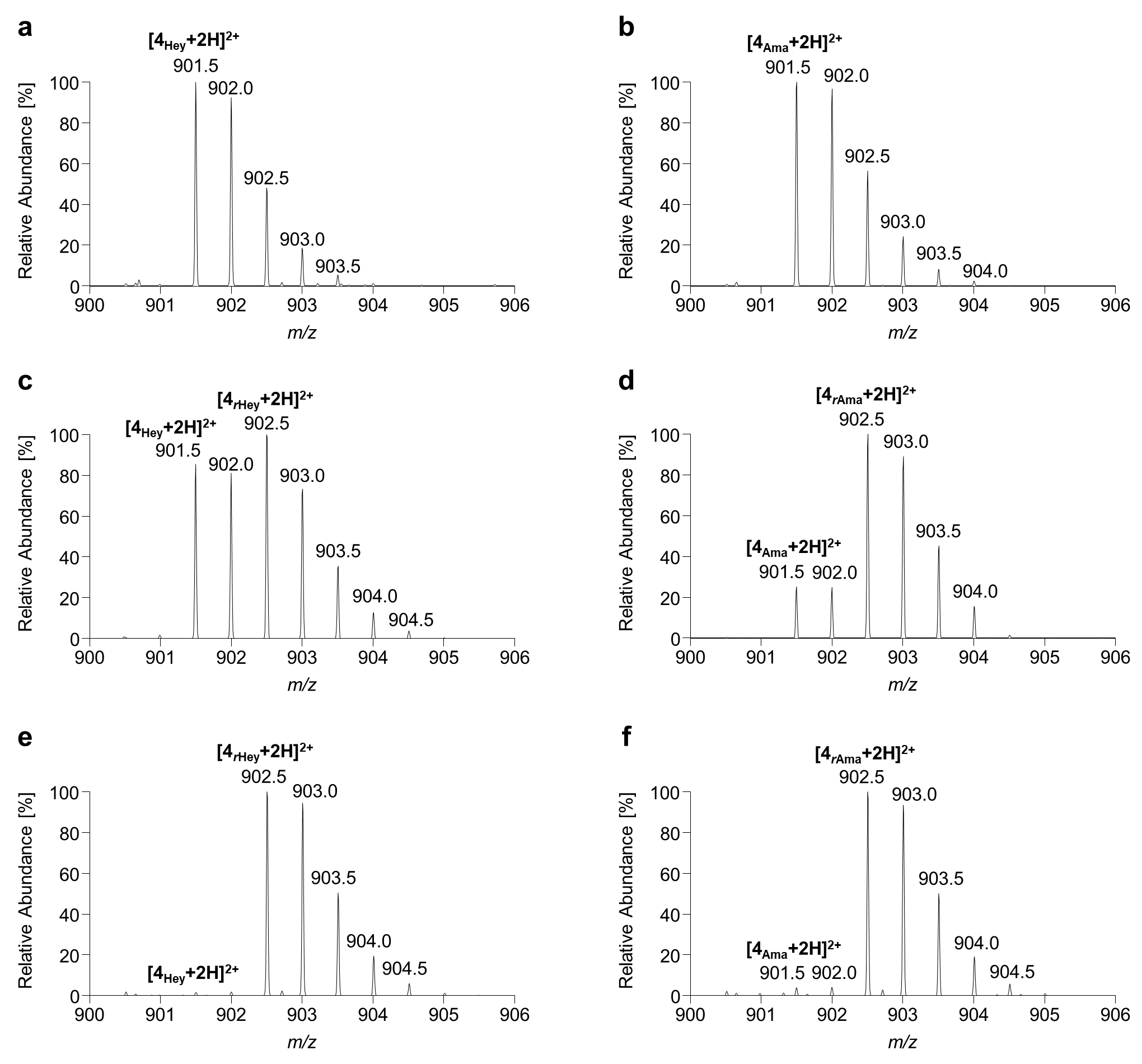


## Fig. S6 Zoomed high-resolution mass spectra (*m/z* 900-906) of two peptide mixtures containing different fructated (Hey) and glucated peptides (Ama) incubated in ammonium acetate (c/d, 250 mmol/L, 50 mmol/L magnesium acetate, pH 8.1) or sodium phosphate loading buffer (e/f, 50 mmol/L, pH 8.5) in the absence (a/b) or presence (*r*Ama/*r*Hey, c-f) of sodium borohydride.

Ratios of signals corresponding to the doubly charged precursor ion of the hexose- (Hey/Ama, *m/z* 901.5) and hexitol-modified (*r*Hey/*r*Ama, *m/z* 902.5) peptide #4 (KVPQVSTPTLVEVSR) represent the efficiency of borohydride reduction.


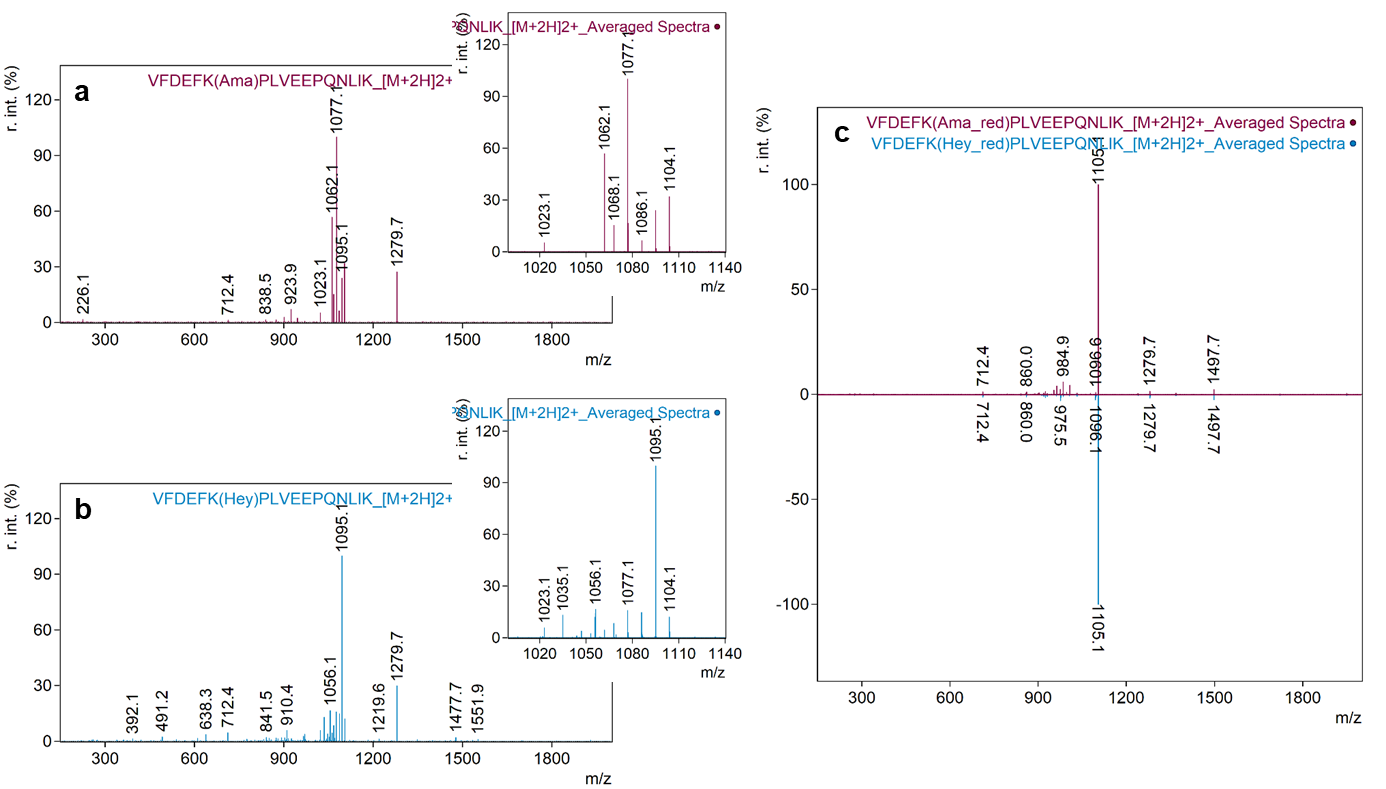


## Fig. S7 Tandem mass spectra of doubly protonated precursor ions of the unreduced (a/b, *m/z* 1104.1) and reduced (c, *m/z* 1105.1) Amadori (red) or Heyns peptide #5 (blue) using a normalized collision energy (NCE) of 22.

The insets show zoomed mass ranges including the characteristic neutral loss pattern of unreduced glycated peptides.


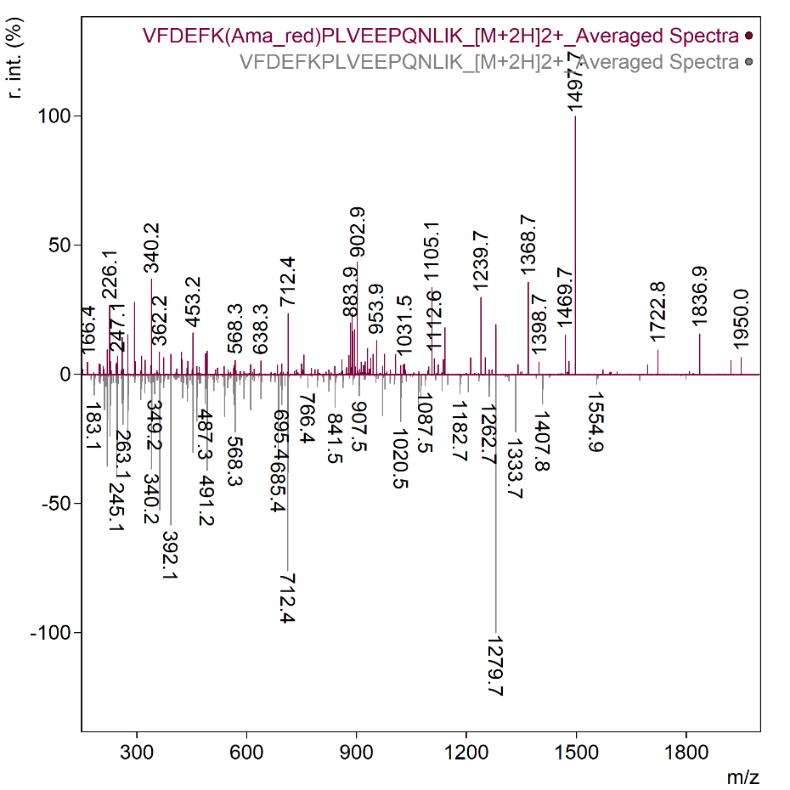


## Fig. S8 Tandem mass spectra of doubly protonated precursor ions of the reduced Amadori (red, *m/z* 1105.1) and the unmodified peptide #5 (gray, *m/z* 1023.1) at a normalized collision energy (NCE) of 30.


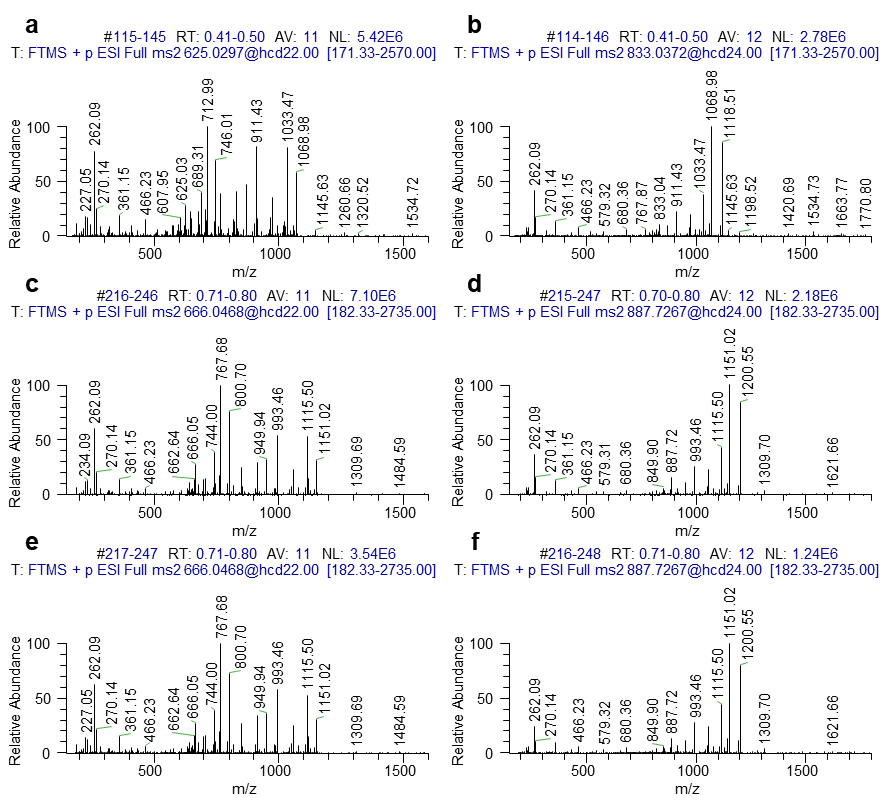


## Fig. S9 Tandem mass spectra recorded for quadruply charged precursor ions detected at *m/z* 625.0297 (a) and *m/z* 666.0468 (c,e) and triply charged precursor ions detected at *m/z* 833.0372 (b) and *m/z* 887.7267 (d,f) of unmodified (a,b), reduced fructated (c,d), and reduced glucated peptide #2 (e,f).


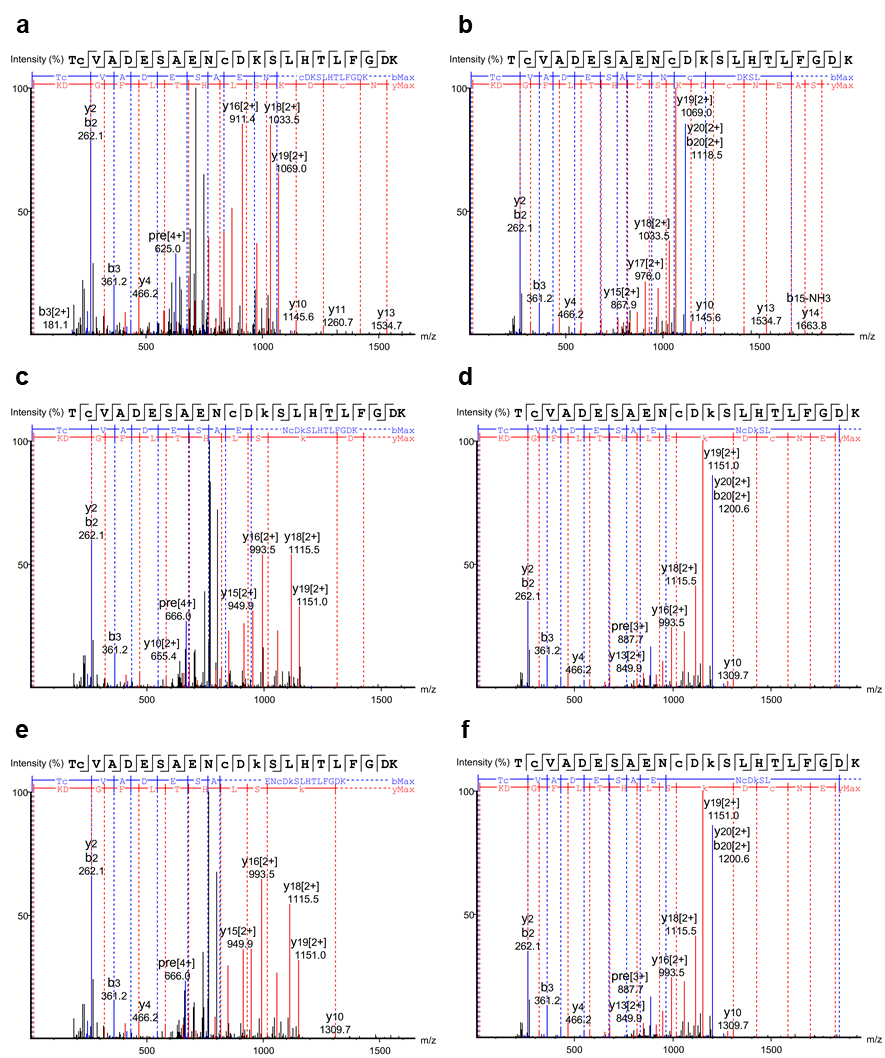


## Fig. S10 Annotated tandem mass spectra recorded for quadruply charged precursor ions detected at *m/z* 625.0297 (a) and *m/z* 666.0468 (c,e) and triply charged precursor ions detected at *m/z* 833.0372 (b) and *m/z* 887.7267 (d,f) of unmodified (a,b), reduced fructated (c,d), and reduced glucated peptide #2 (e,f).


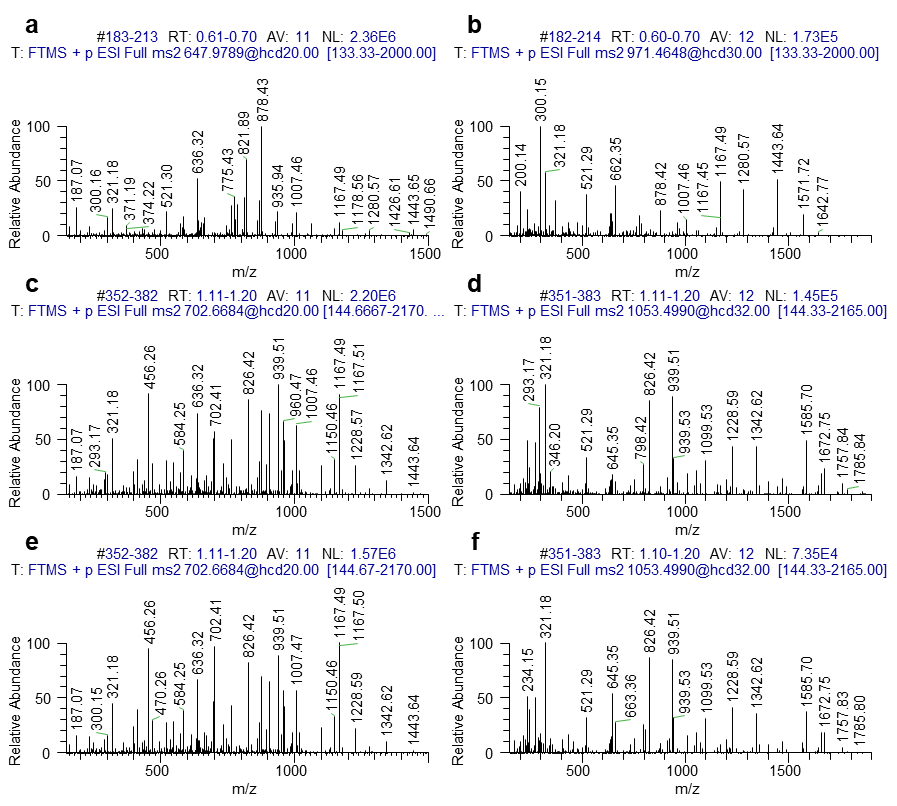


## Fig. S11 Tandem mass spectra recorded for triply charged precursor ions detected at *m/z* 647.9789 (a) and *m/z* 702.6684 (c,e) and doubly charged precursor ions detected at *m/z* 971.4648 (b) and *m/z* 1053.4990 (d,f) of unmodified (a,b), reduced fructated (c,d), and reduced glucated peptide #3 (e,f).


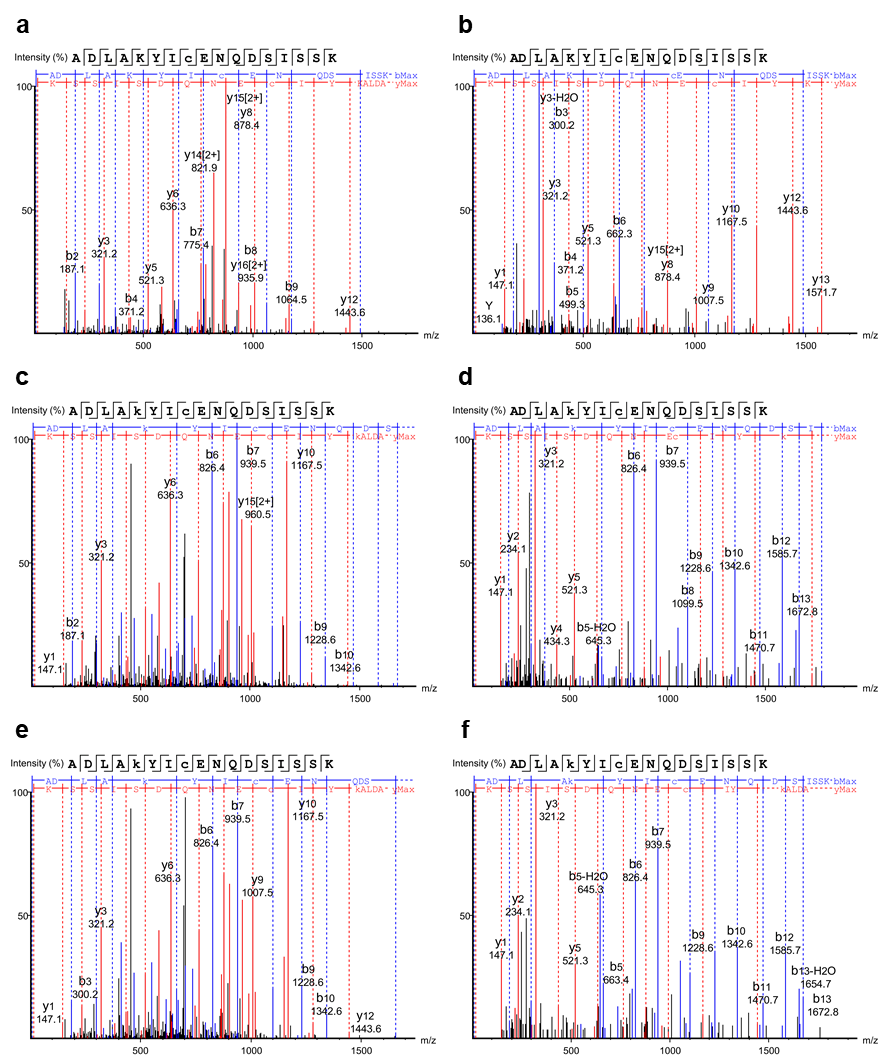


## Fig. S12 Annotated tandem mass spectra recorded for triply charged precursor ions detected at *m/z* 647.9789 (a) and *m/z* 702.6684 (c,e) and doubly charged precursor ions detected at *m/z* 971.4648 (b) and *m/z* 1053.4990 (d,f) of unmodified (a,b), reduced fructated (c,d), and reduced glucated peptide #3 (e,f).


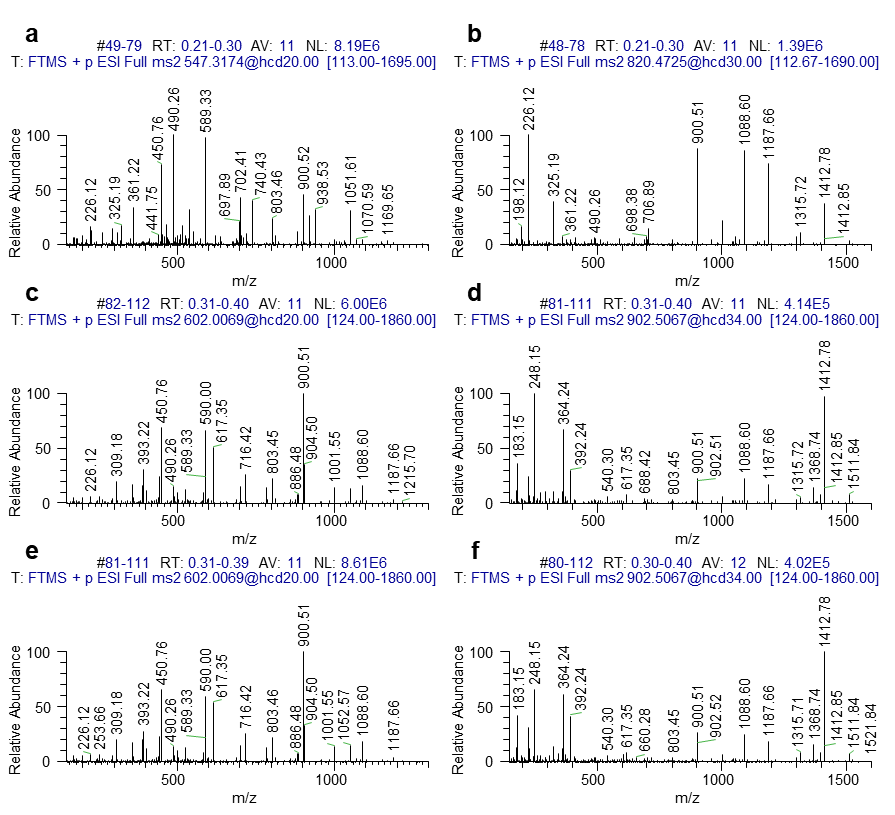


## Fig. S13 Tandem mass spectra recorded for triply charged precursor ions detected at *m/z* 547.3174 (a) and *m/z* 602.0069 (c,e) and doubly charged precursor ions detected at *m/z* 820.4725 (b) and *m/z* 902.5067 (d,f) of unmodified (a,b), reduced fructated (c,d), and reduced glucated peptide #4 (e,f).


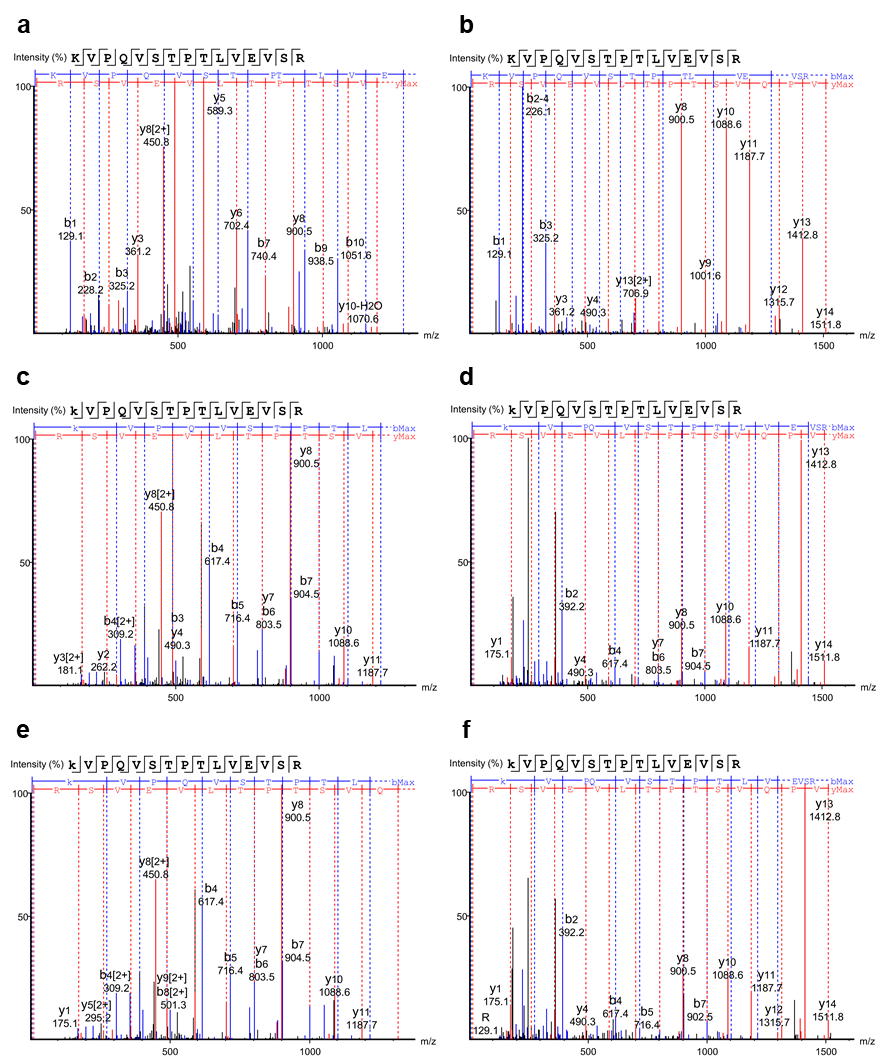


## Fig. S14 Annotated tandem mass spectra recorded for triply charged precursor ions detected at *m/z* 547.3174 (a) and *m/z* 602.0069 (c,e) and doubly charged precursor ions detected at *m/z* 820.4725 (b) and *m/z* 902.5067 (d,f) of unmodified (a,b), reduced fructated (c,d), and reduced glucated peptide #4 (e,f).


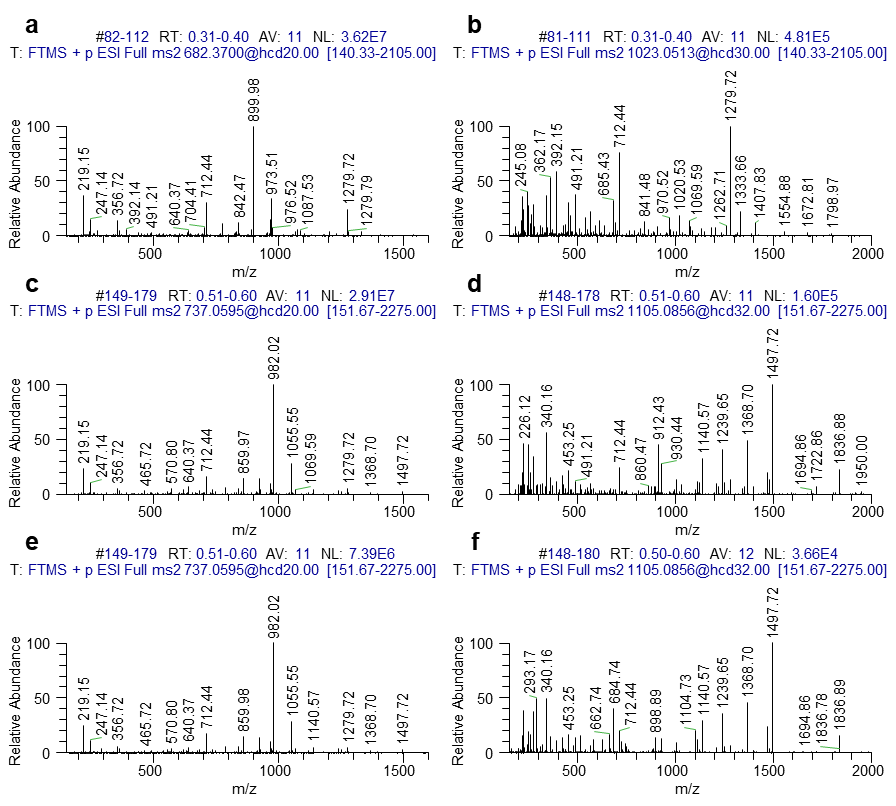


## Fig. S15 Tandem mass spectra recorded for triply charged precursor ions detected at *m/z* 682.3700 (a) and *m/z* 737.0595 (c,e) and doubly charged precursor ions detected at *m/z* 1023.0513 (b) and *m/z* 1105.0856 (d,f) of unmodified (a,b), reduced fructated (c,d), and reduced glucated peptide #5 (e,f).


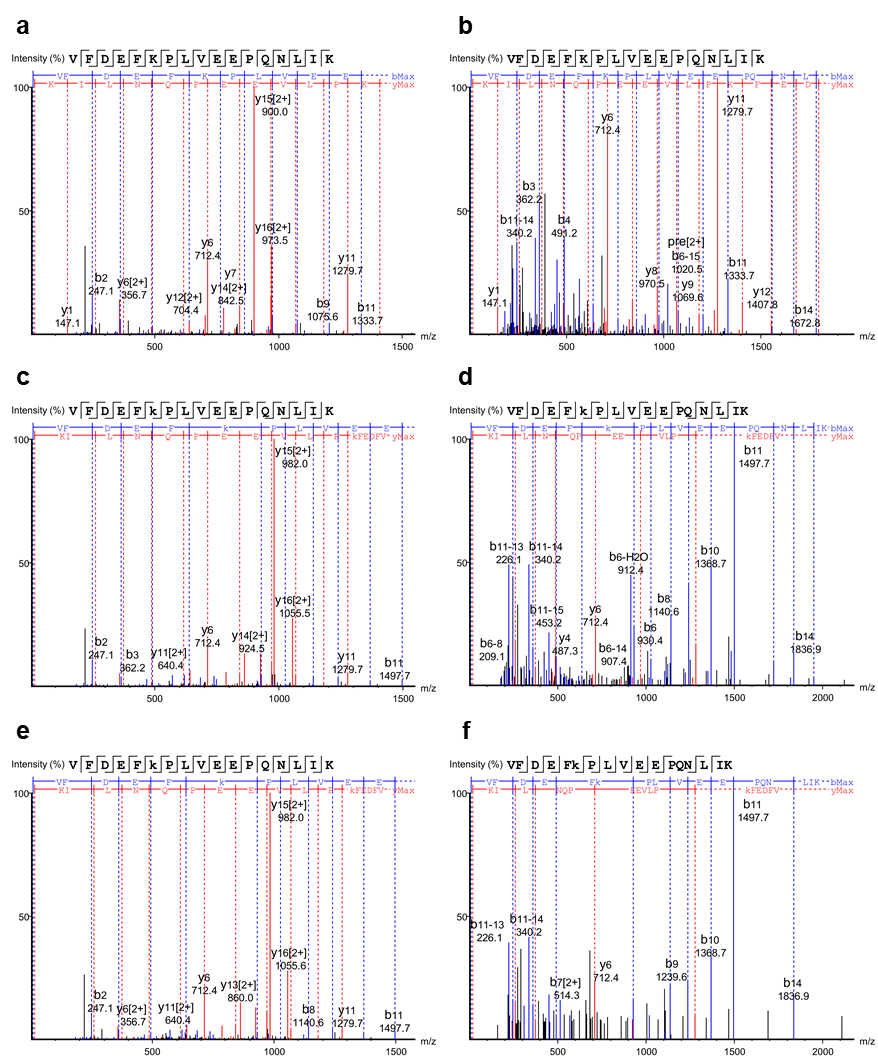


## Fig. S16 Annotated tandem mass spectra recorded for triply charged precursor ions detected at *m/z* 682.3700 (a) and *m/z* 737.0595 (c,e) and doubly charged precursor ions detected at *m/z* 1023.0513 (b) and *m/z* 1105.0856 (d,f) of unmodified (a,b), reduced fructated (c,d), and reduced glucated peptide #5 (e,f).


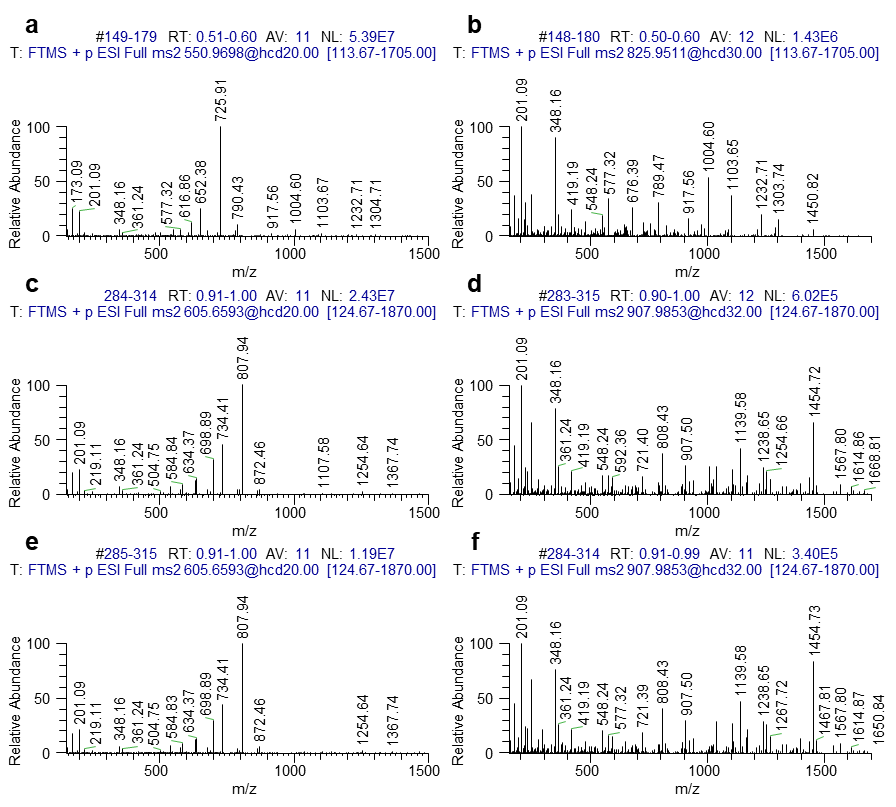


## Fig. S17 Tandem mass spectra recorded for triply charged precursor ions detected at *m/z* 550.9698 (a) and *m/z* 605.6593 (c,e) and doubly charged precursor ions detected at *m/z* 825.9511 (b) and *m/z* 907.9853 (d,f) of unmodified (a,b), reduced fructated (c,d), and reduced glucated peptide #6 (e,f).


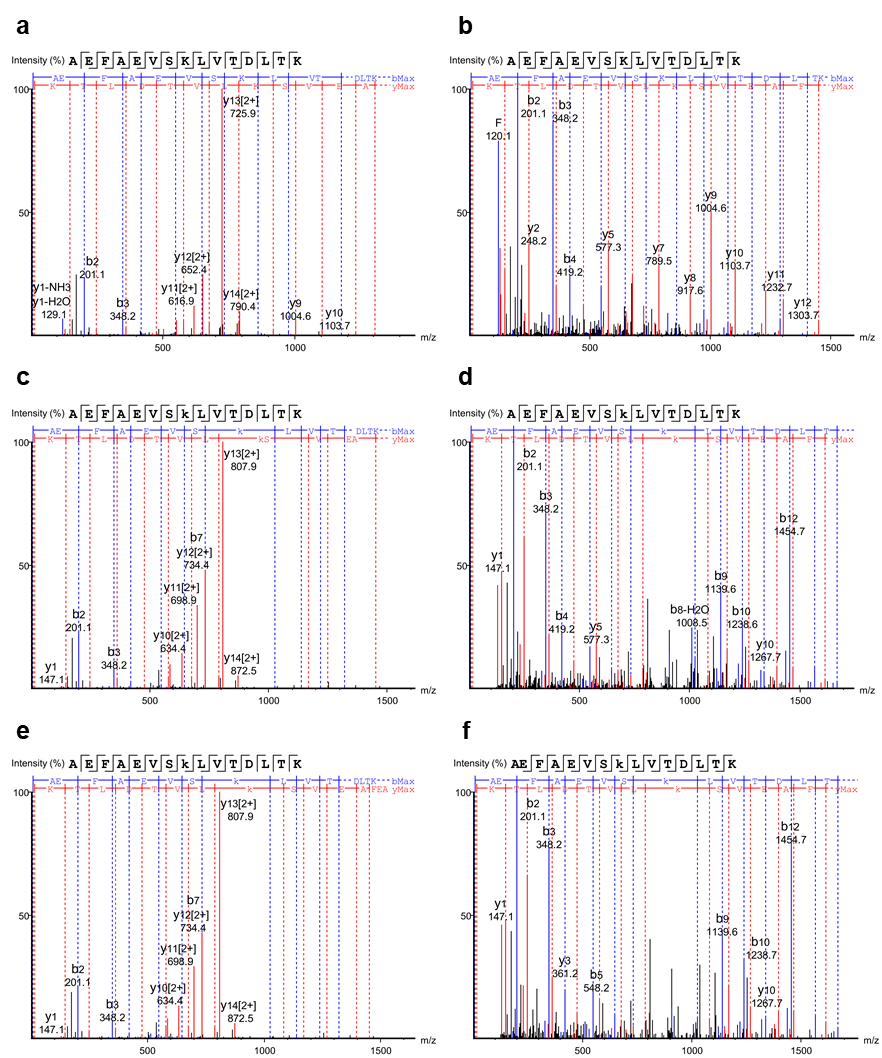


## Fig. S18 Annotated tandem mass spectra recorded for triply charged precursor ions detected at *m/z* 550.9698 (a) and *m/z* 605.6593 (c,e) and doubly charged precursor ions detected at *m/z* 825.9511 (b) and *m/z* 907.9853 (d,f) of unmodified (a,b), reduced fructated (c,d), and reduced glucated peptide #6 (e,f).


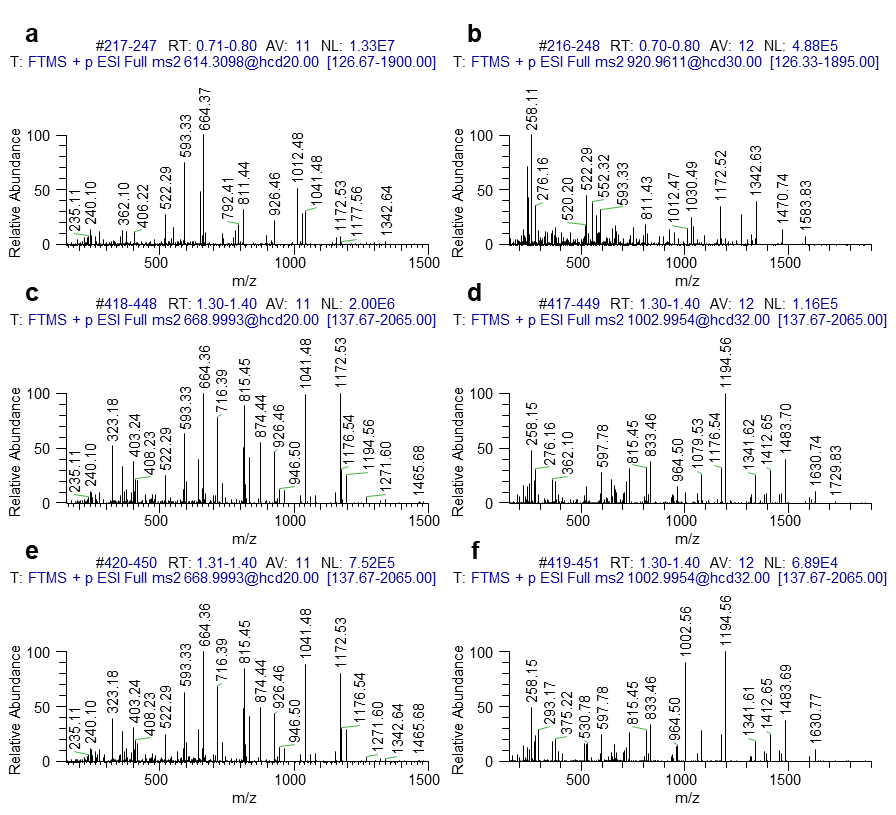


## Fig. S19 Tandem mass spectra recorded for triply charged precursor ions detected at *m/z* 614.3098 (a) and *m/z* 668.9993 (c,e) and doubly charged precursor ions detected at *m/z* 920.9611 (b) and *m/z* 1002.9954 (d,f) of unmodified (a,b), reduced fructated (c,d), and reduced glucated peptide #7 (e,f).


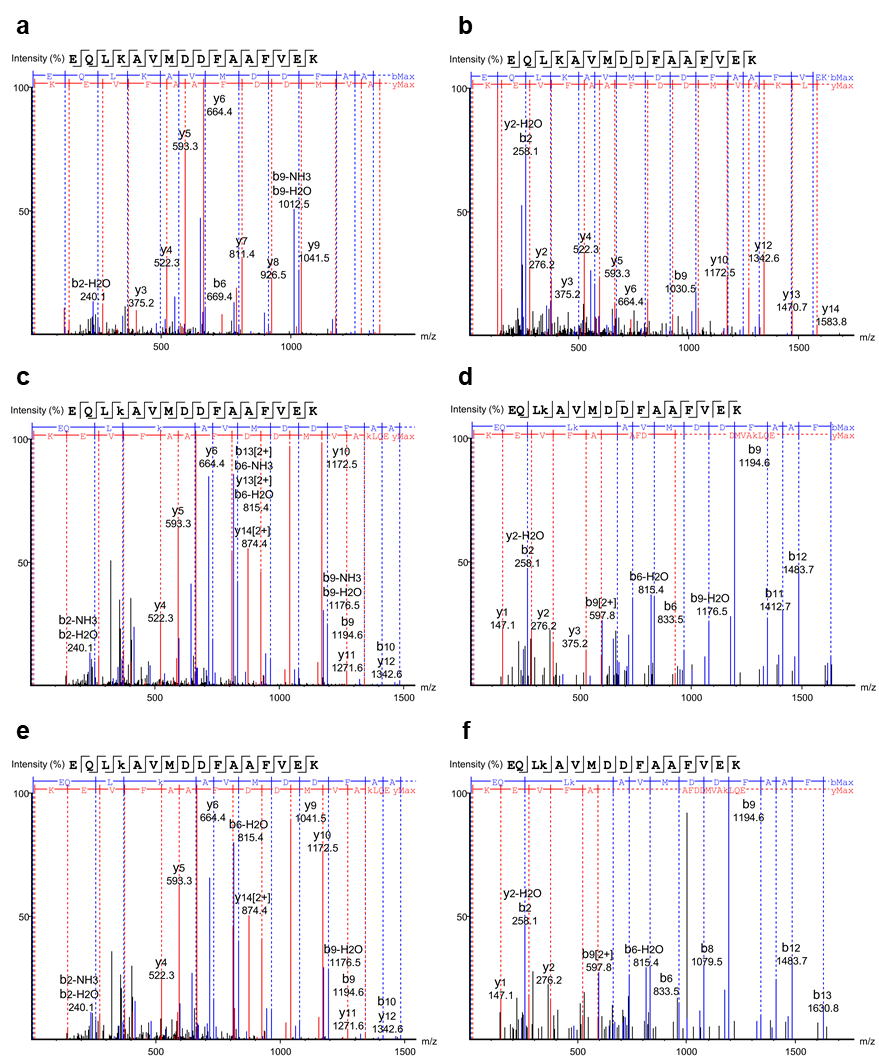


## Fig. S20 Annotated tandem mass spectra recorded for triply charged precursor ions detected at *m/z* 614.3098 (a) and *m/z* 668.9993 (c,e) and doubly charged precursor ions detected at *m/z* 920.9611 (b) and *m/z* 1002.9954 (d,f) of unmodified (a,b), reduced fructated (c,d), and reduced glucated peptide #7 (e,f).


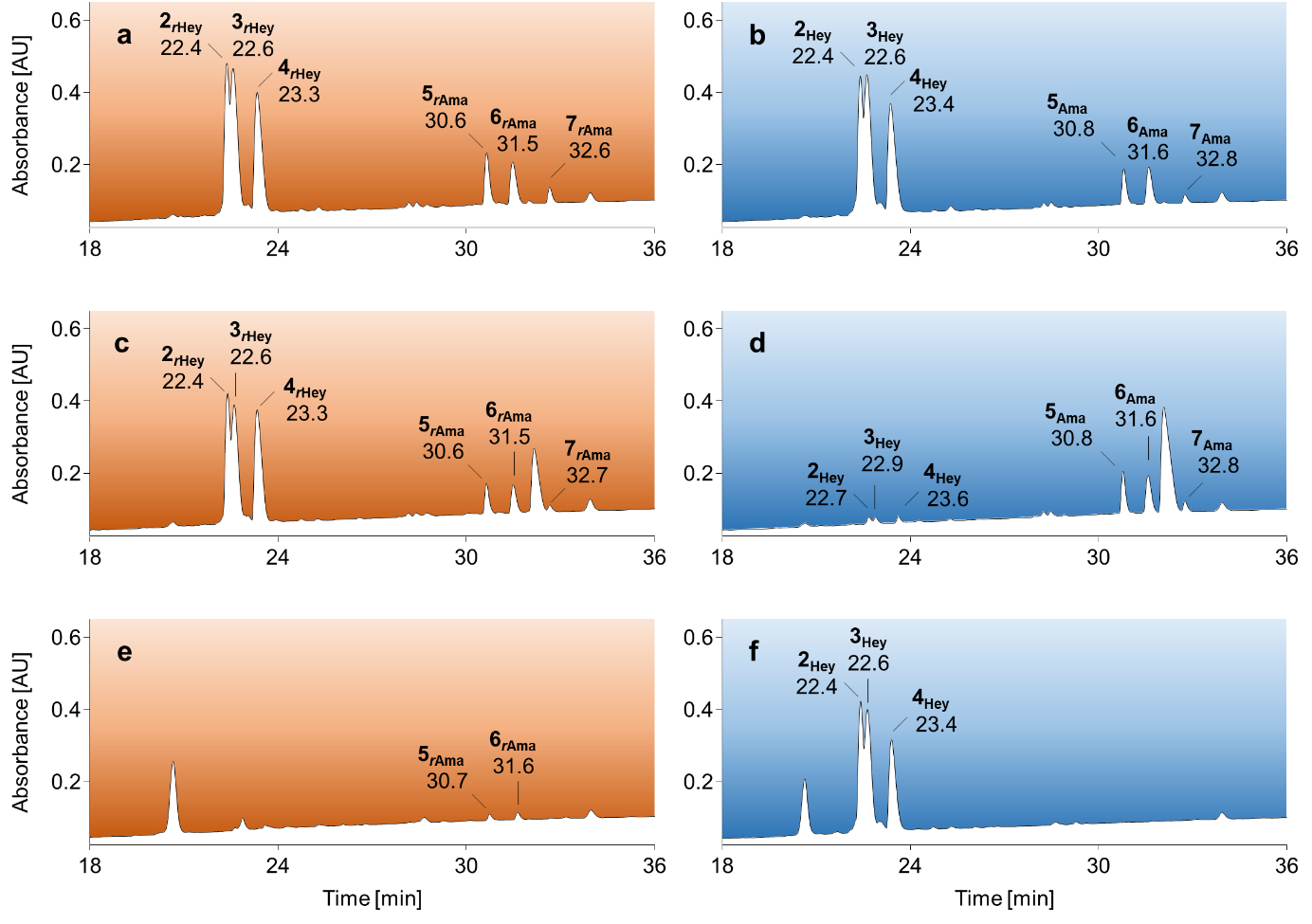


## Fig. S21 RP chromatograms from 18 to 36 min of an SPE-purified peptide mixture containing three fructated (Hey) and three glucated peptides (Ama, 1 nmol each) incubated in sodium phosphate buffer (50 mmol/L, pH 8.5) in the presence (orange, *r*Ama/*r*Hey, a) or absence (blue, b) of sodium borohydride and the corresponding elution (c,d) and wash fractions (e,f) obtained by BAC using ammonium acetate loading buffer (250 mmol/L, 50 mmol/L magnesium acetate, pH 8.1).

Peptides were separated on an Aqua C_18_-column at 60°C using a linear gradient from 97% eluent A to 40% eluent C in 37 min (absorbance recorded at 214 nm). Peptide sequences and modification sites are listed in Tab. S3. Results for the complementary peptide mixture are shown in Fig. S21. Recovery rates and percentage distribution in affinity processed fractions are summarized in Tab. S5 and Tab. S6 (Exp 7).

**
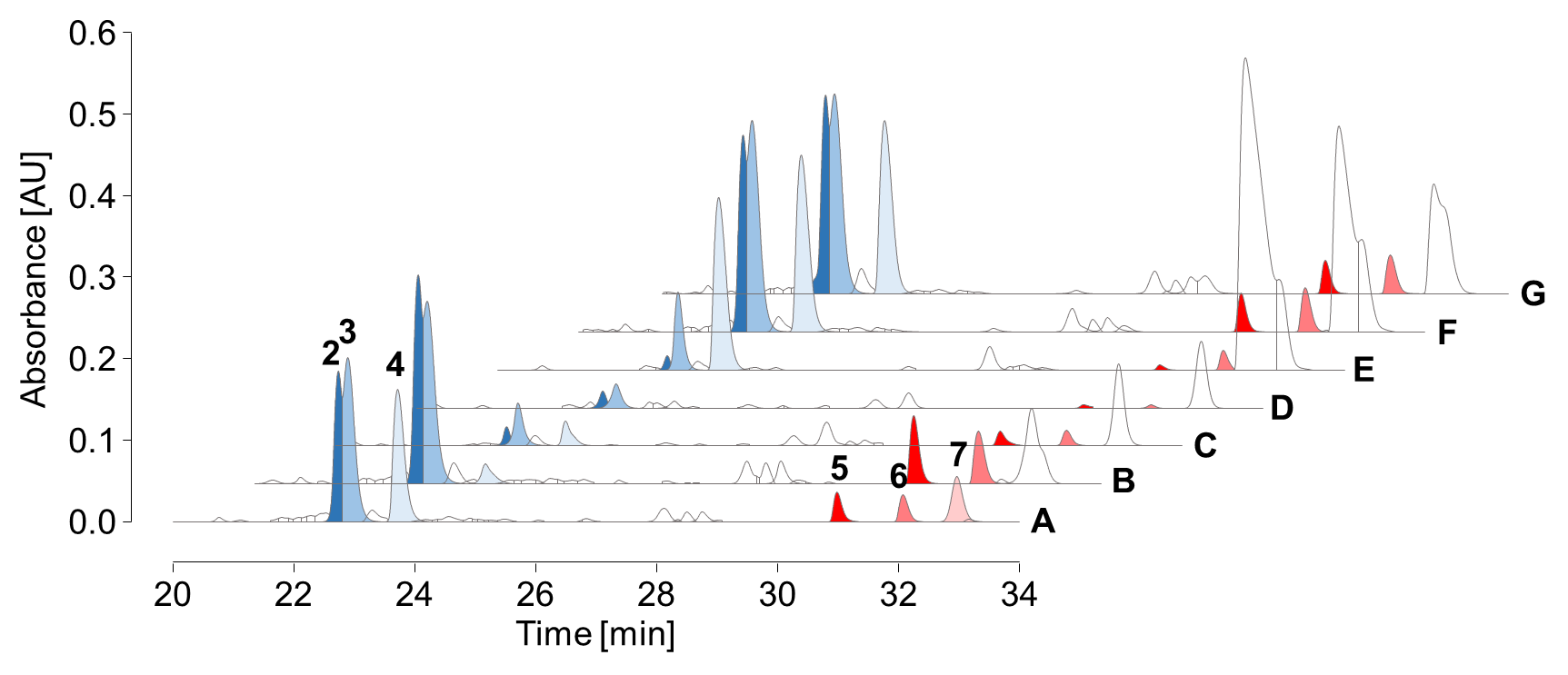
**

## Fig. S22 RP chromatograms from 20 to 34 min of an SPE-purified peptide mixture (A) containing three fructated (Heyns) and three glucated peptides (Amadori, *n*=3, 1 nmol each) reduced with sodium borohydride and the wash (B-D) and enriched fractions (E-G) collected by BAC.

Equilibration, sample loading, and washing in BAC were performed using either sodium phosphate (pH 8.5, B,E) or ammonium acetate loading buffer (pH 8.1, C,F), the latter also with additional equilibration and sample loading at pH 10 (D/G). Peptides were separated on an Aqua C_18_-column at 60°C using a linear gradient from 97% eluent A to 40% eluent C in 37 min. The absorbance was recorded at 214 nm. Peptide sequences and modification sites are listed in Tab. S3. Recovery rates and percentage distribution in affinity processed fractions are summarized in Tab. S5 and Tab. S6 (Exp 8/Exp 9/Exp 10).
